# Biochar-Derived Persistent Free Radicals: A Plethora of Environmental Applications in a Light and Shadows Scenario

**DOI:** 10.3390/toxics12040245

**Published:** 2024-03-27

**Authors:** Silvana Alfei, Omar Ginoble Pandoli

**Affiliations:** 1 Department of Pharmacy (DIFAR), University of Genoa, Viale Cembrano 4, 16148 Genoa, Italy; omar.ginoblepandoli@unige.it; 2 Department of Chemistry, Pontifical Catholic University, Rua Marquês de São Vincente 225, Rio de Janeiro 22451-900, Brazil

**Keywords:** biochar (BC), pyrolysis, biochar-derived permanent free radicals (PFRs), reactive oxygen species (ROS), PFR-mediated BC applications, environmental risk

## Abstract

Biochar (BC) is a carbonaceous material obtained by pyrolysis at 200–1000 °C in the limited presence of O_2_ from different vegetable and animal biomass feedstocks. BC has demonstrated great potential, mainly in environmental applications, due to its high sorption ability and persistent free radicals (PFRs) content. These characteristics enable BC to carry out the direct and PFRs-mediated removal/degradation of environmental organic and inorganic contaminants. The types of PFRs that are possibly present in BC depend mainly on the pyrolysis temperature and the kind of pristine biomass. Since they can also cause ecological and human damage, a systematic evaluation of the environmental behavior, risks, or management techniques of BC-derived PFRs is urgent. PFRs generally consist of a mixture of carbon- and oxygen-centered radicals and of oxygenated carbon-centered radicals, depending on the pyrolytic conditions. Here, to promote the more productive and beneficial use of BC and the related PFRs and to stimulate further studies to make them environmentally safer and less hazardous to humans, we have first reviewed the most common methods used to produce BC, its main environmental applications, and the primary mechanisms by which BC remove xenobiotics, as well as the reported mechanisms for PFR formation in BC. Secondly, we have discussed the environmental migration and transformation of PFRs; we have reported the main PFR-mediated application of BC to degrade inorganic and organic pollutants, the potential correlated environmental risks, and the possible strategies to limit them.

## 1. Introduction

Biochar (BC) is a stable carbon-rich black solid substance produced from vegetable or animal biomass feedstocks when pyrolyzed. Pyrolysis is a procedure that involves the heating of substrates at 200–1000 °C under oxygen-limited conditions [1]. The term “biochar” derives from the combination of “bio-,” which stands for “biomass,” and “char,” meaning “charcoal.” In recent years, BC has received widespread attention due to its potential application in carbon sequestration, soil amendment/remediation, wastewater treatment, and catalysis [2,3,4,5,6,7,8,9,10,11]. In particular, several ground-breaking studies have been carried out to investigate the potential of BC in alternative energy production and in the recovery of value-added chemicals/by-products [3]. In this regard, Lee et al. have used BC as briquettes and electrodes for microbial fuel cells (MFCs) finalized for alternative energy production [5]. Zhang et al. have employed BC as an additive/catalyst in anaerobic digestion and transesterification reactions for biogas [6], while Behera et al. employed BC to produce biodiesel [4]. Environmental applications of BC for reducing gaseous emissions, mainly by carbon and nitrogen sequestration, are also gaining attention [8].

**Figure 1:**
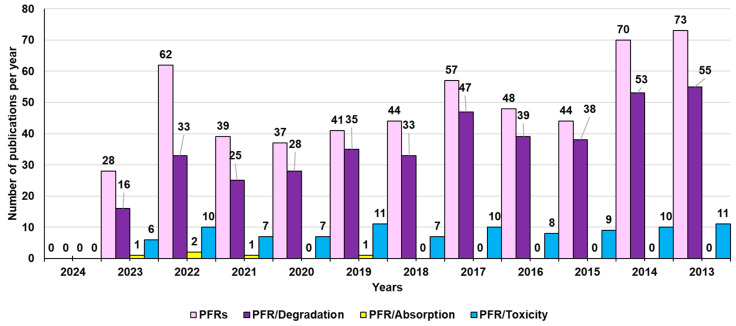
Number of publications on PFRs and their degradation, absorption, and possible toxic actions published over the last ten years (current year excluded) according to the PubMed dataset. The survey was carried out using the following keywords: permanent free radicals (pink bars); permanent free radicals AND degradation (purple bars); permanent free radicals AND absorption (yellow bars); permanent free radicals AND toxicity (light blue bars).

BC has been applied to prevent eutrophication by recovering excessive nutrients, including nitrogen and phosphorus, from wastewater [11], while the agronomic application of BC, due to its characteristics of high cation exchange capacity (CEC) and specific surface area (SSA), have recently been reported by Zhao et al. [10]. Batch and column sorption experiments have shown that certain types of BC have good adsorption performance for heavy metals, dyes, or phosphate from aqueous solutions and are being investigated as cost-effective, promising, and eco-friendly alternative adsorbent materials [12]. Additionally, BC has demonstrated high efficiency in removing pharmaceuticals [13], pesticides [14], polycyclic aromatic hydrocarbons (PAHs) [15], and petroleum derivatives [16]. Also, the metal ion absorbent capacity of BC has been extensively reported in both the absence and presence of fulvic acid and humic acid [17]. As a soil improver, BC can reduce soil acidity and help maintain soil moisture and nutrient levels. Through its carbon sequestration action, BC performs climate restoration. Moreover, due to its strong adsorption capacity, BC can remove environmental xenobiotics, thus preventing their uptake in plants, animals, and humans [18,19,20,21]. Additionally, BC derived from the thermal treatment of organic material generally contains persistent free radicals (PFRs) bound to the external or internal surfaces of its solid particles [22,23]. Such BC-bound PFRs, which are reactive due to unpaired electrons, can persist for minutes and up to several months, in contrast to traditional transient radicals [24], thus conferring on BC the capacity to degrade organic pollutants through the generation of other reactive oxygen species (ROS) and sulfate radicals [25,26,27]. In this context, BC-bound PFRs have been investigated to activate persulfate (S_2_O_8_^2−^) to obtain sulfate radicals, which have efficiently degraded phenolic compounds and polychlorinated biphenyls [28], acid orange 7 [29], and sulfamethoxazole [30,31]. In the presence of PFRs, hydrogen peroxide (H_2_O_2_) or oxygen (O_2_) have been activated to produce hydroxyl radicals (OH•) and superoxide radicals (O_2_•^−^), which succeeded in efficiently degrading chloro-biphenyl [32], diethyl phthalate [33,34], and ciprofloxacin [35]. By the PFR-mediated activation of peroxyl mono sulfate (PMS), radical species such as SO_4_•^−^, •OH, and O_2_•^−^, as well as non-radical species such as ^1^O_2_ formed, which were the main contributors in the antibiotics’ degradation [36]. On the other hand, by stimulating the production of ROS, PFRs can inhibit seed germination and retard the growth of roots and shoots [37]. Additionally, BC production itself may cause the release of xenobiotics such as polycyclic aromatic hydrocarbons (PAHs), toxic inorganic elements, and dioxins, thus posing potential risks to human health and the environment [1]. The scientific community should evaluate BC and BC-bound PFRs’ positive and negative impacts before their extensive ecological application. Although the environmental behavior and risks of BC and BC-associated PRFs are increasingly attracting research attention, in the last ten years (the year 2024 excluded), studies into their toxicity remain very limited (96 publications), compared to those concerning PFRs in general (542 publications) and their degradation capability (402 publications) (Figure 1).

**Figure 2:**
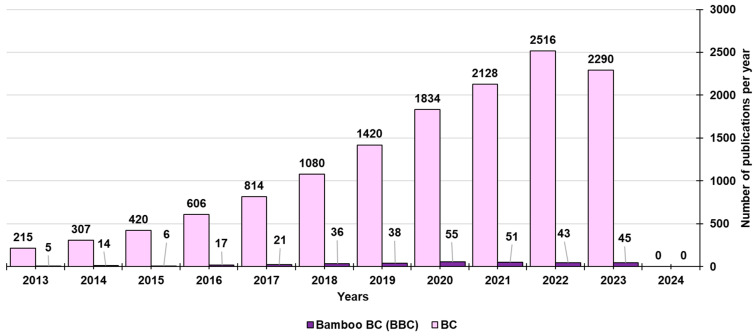
Number of publications on BCs and on bamboo-derived BCs published in the last ten years (current year excluded) according to the PubMed dataset. The survey used the following keywords: biochar (pink bars) and bamboo biochar (purple bars).

However, to safeguard the environment from BC-related PFRs’ potential adverse effects, it is necessary to comprehensively and systematically consider their environmental risks, formation mechanisms, and controlling factors [27], as well as the corresponding possible mitigation actions. Studies have shown that the type of biomass feedstock used to produce BC is pivotal in determining the physicochemical properties of the resulting BC, which also strongly affect the formation and characteristics of PFRs. To date, the types of biomasses used to prepare BC and involved in the investigation of BC-bound PFRs mainly include lignocellulosic biomasses (hemicellulose, cellulose, and lignin) from sources such as pine needles, wheat straw, lignin, cow manure, rice husk, and maize straw [38,39,40]. Additionally, cow dung (CD), sheep manure (SM), lotus stem (LS), and eggshell (ES) biomasses, representative of farm wastes, have been reported to provide BC containing PFRs [41]. Bamboo is an emerging starting material that is perfect for synthesizing BC and activated carbon (AC) due to its inexpensive cost, high biomass yield, and accelerated growth rate [42,43]. However, only a few researchers and scientists have used bamboo as a unique source for developing BC so far, as established by the number of publications on bamboo-derived BC developed in the last ten years (current year excluded) (331) vs. those on BC derived from different sources (13,630) (Figure 2).

**Figure 3:**
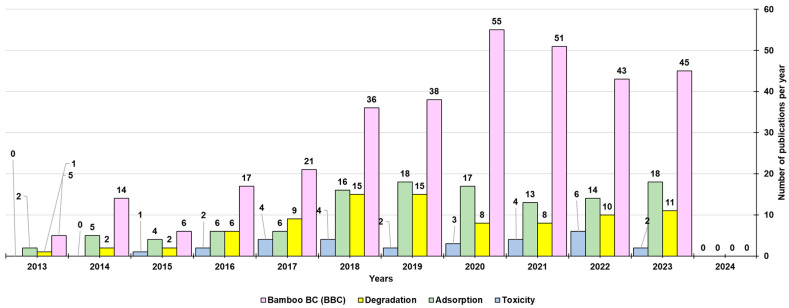
Number of publications on bamboo-derived BCs, as well as their absorption, degradation, and toxic properties, published in the last ten years (current year excluded) according to the PubMed dataset. The survey was carried out using the following keywords: bamboo biochar (pink bars); bamboo biochar AND adsorption (green bars); bamboo biochar AND degradation (yellow bars); bamboo biochar AND toxicity (light blue bars).

Among the 331 publications on bamboo-derived BC, most were about their adsorption activity (119), followed by those on their degradation capacity (87). At the same time, few studies were conducted on their possible toxic action (Figure 3).

In this scenario, to promote the more productive and beneficial use of BC and the related PFRs and stimulate further studies to make them environmentally safer and less hazardous to humans, we have first reviewed the most common methods used to produce BC and its main environmental applications, as well as the reported mechanisms for PFR formation in BC. The main factors influencing the physicochemical properties of BC have also been reported. Secondly, we have discussed the environmental migration and transformation of PFRs, the main PFR-mediated applications of BC to remediate inorganic and organic pollutants described in the last five years, the correlated potential risks to the environment and humans, and the possible strategies to limit them. To confirm the relevance and essentiality of the present paper, a recent survey of the PubMed dataset has evidenced that, although the number of studies on BC-related PFRs has increased in the last few years, they are still very limited compared to those on BC in general (134 vs. 11646 from 2014 to the present). Additionally, the review articles on BC-associated PFRs that, by gathering information on the topic, could stimulate more research on it are indeed limited (16). Some recent examples could be considered works by Zhang et al., Liu et al., Luo et al., and Yi et al. [1,44,45,46]. In this scenario, this review can be considered essential because it offers an all-round and complete overview of both BC and BC-related PFRs, via an extensive discussion on both their beneficial impact and the possible risks to humans and the environment that could derive from their widespread and indiscriminate application. In the case of the present paper, we have provided a reader-friendly work where the information has mostly been organized into easy-to-read tables, schemes, and statistical graphs that could have a profound impact on its readers’ understanding.

## 2. Biochar (BC)

The constant growth in world population translates into a continued increase in the global energy requirement by all sectors and a dramatic decrease in fossil fuels, the primary energy source [47,48,49]. Furthermore, the effect of the resulting CO_2_ emissions on the environment determines additional global energy issues, which make the replacement of fossil fuels necessary and urgent [50]. In this regard, biochar (BC), mainly obtained from organic waste and possessing the capability of sequestering carbon, represents a rich carbon source and an alternative to fossil fuels [51,52]. Table S1 in the Supplementary Materials reports the biomasses commonly used for BC production [53,54,55,56,57,58,59,60,61,62,63,64,65,66].

**Figure 4:**
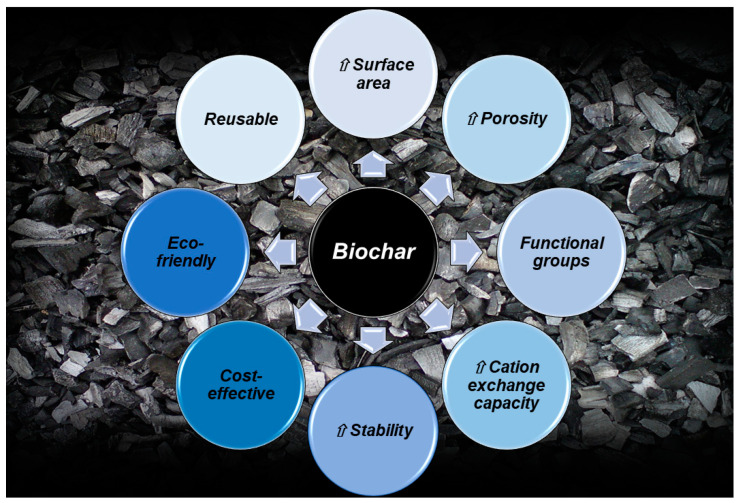
Some of the advantages and physicochemical properties of BCs. ⇑ = high.

BC obtained by the combustion of various biomasses, as reported in Table S1, has been demonstrated to possess unparalleled physicochemical properties such as a large surface area, high porosity, the presence of several functional groups, high cation exchange capacity (CEC), long-term stability, etc. (Figure 4). Such properties make BC suitable for various applications, including, but not limited to, carbon sequestration, soil amendment, energy storage, and catalysis [67,68,69,70,71,72,73,74,75] (Table S1). Additionally, BC is cost-effective, has an eco-friendly nature, and is endowed with reusability (Figure 4) [76,77]. Mainly, BC is increasingly gaining attention from many researchers as a material to efficiently remove various environmental contaminants, including antibiotics, thus reducing the emergence of microbial resistance [72,74].

Among the biomass waste materials appropriate for BC production, crop residues from agriculture, forestry, municipal solid waste, food, and animal manure have high potential [78,79,80,81,82,83].

### 2.1. Main Methods of Producing Biochar

As reported in the following Table 1, BC can be prepared rapidly using thermochemical conversion techniques such as pyrolysis, hydrothermal carbonization, gasification, flash carbonization, and torrefaction [84,85], with pyrolysis the most widely adopted (Section 2.1.1).

#### 2.1.1. Pyrolysis

**Figure 5:**
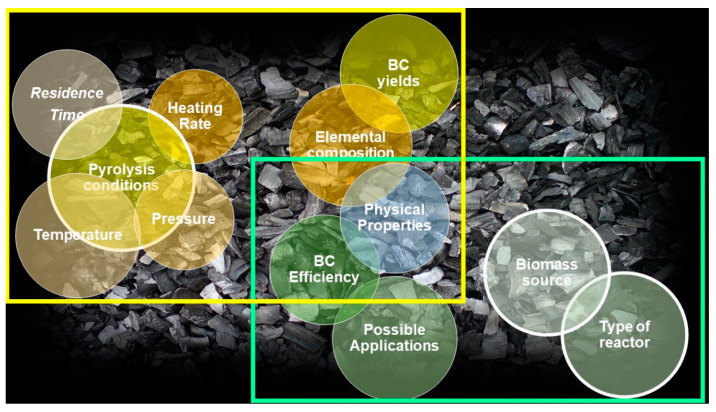
Parameters that mainly influence the pyrolysis process outcomes and the prepared BC elemental composition, physicochemical properties, and possible applications.

Pyrolysis is a thermochemical process wherein the organic compounds present in the biomass are decomposed at a specific temperature [91]. Mainly, during pyrolysis, the thermal decomposition of organic materials occurs in an oxygen-free or oxygen-limited environment within a temperature range of 250–1000 °C [92]. In these conditions, the lignocellulosic components of biomass, such as cellulose, hemicellulose, and lignin, go through chemical reactions like depolymerization, fragmentation, and cross-linking, depending on the adopted temperatures. There are three principal possible products, including solid, liquid, and gas physical state materials. The solid products comprise BC and ash, while the liquid ones encompass bio-oils and tar, and the gaseous products (syngas) comprise carbon dioxide, carbon monoxide, hydrogen, and C1-C2 hydrocarbons [86]. As shown in Figure 5, during pyrolysis, the process parameters, including temperature, the type and nature of the biomass, residence time, heating rate, pressure, etc., could strongly affect BC yield and its physicochemical characteristics [93,94]. Moreover, although BC samples derived from different biomasses are all entirely made of carbon content and ash, their elemental composition, as well as their physical characteristics and properties, could differ enormously based on the type of biomass, reaction conditions, and type of reactor used during the carbonization process [95] (Figure 5). Consequently, every experimental condition and the starting raw material should be considered as a proof-of-concept of the future industrial application of BC.

The most widely used reactors for the chemical transformation of different biomasses include paddle kilns, bubbling fluidized beds, wagon reactors, tubular ovens, and agitated sand rotary kilns. However, temperature remains the primary operating process condition that governs the yield in BC, vs. those of the oily and gaseous products. Usually, BC yield decreases and syngas production increases when the pyrolysis temperature is improved [96]. Based on the heating rate, temperature, residence time, and pressure, pyrolysis can be categorized as fast or slow, as summarized in Table S2 in the Supplementary Materials [97]. Generally, fast pyrolysis is employed to maximize the liquid product yield, while slow pyrolysis is employed to maximize the solid product yield [98].

### 2.2. Biochar Characterization and Main Properties

The characterization of BC to determine its elemental composition is carried out by performing elemental analyses. Otherwise, its physicochemical, surface, and structural characterization is carried out by determining its surface functional groups, stability, and structure by employing various modern techniques reported in Table S3 in the Supplementary Materials [57].

As mentioned, the source of feedstock and the heat treatment temperatures during preparation are two significant factors that determine the physiochemical properties of BC.

The properties of pristine biomass that mainly influence the related BC include moisture content, ash content, calorific value, the percentage of lignin, cellulose, hemicellulose, fractions of fixed carbon, and volatile components [98]. High-yield BC with high porosity is achievable using biomasses possessing more lignin and less cellulose. Additionally, the volatile component, water content, particle size, and shape of the original biomass can also affect the properties of BC [98]. Table 2 reports the general chemical and physical features of BC, while Table S4 in the Supplementary Materials reports some characteristics of BCs produced from specific feedstocks at various production temperatures [99].

#### The Question of Temperature

As already reported, the pyrolysis temperature and feedstock greatly influence the physicochemical properties of BC, including pH, specific surface area, pore size, CEC, volatile matter, ash, and carbon content. CEC and volatile matter decrease with increasing pyrolysis temperature, whereas pH, specific surface area, ash, carbon content, and pore volume increase with an increase in pyrolysis temperature [100]. Increasing temperature also causes a decrease in the number of acidic functional groups, especially carboxylic functional groups, and causes the appearance of carbonylic functional groups and alkalinity [101]. In particular, unpaired negative charges forming during pyrolysis at higher temperatures enable BC to accept protons [101]. Although BC’s alkalinity increases with higher pyrolysis temperatures, thus improving its capacity to neutralize acids in soils, lower temperatures are necessary to preserve functional groups and obtain BC with higher CEC [102]. Low water content in BC, which reduces the possible microbial activity, promoting self-healing and degradation, is achievable at a higher temperature. However, the highly porous structure of BC obtained in such conditions causes the ready adsorption of moisture from the surroundings, thus increasing water content, re-enabling microbial activity, and contributing to self-heating and degradation [100].

During biomass decomposition to BC, the total surface area changes like the porosity due to the escaping of volatile gases and increases with increasing temperature [103]. In this regard, a large surface area affects CEC and water-holding capacity (WHC). Curiously, during pyrolysis, the hydro-properties of the initial biomass undergo several modifications depending on the pyrolysis temperature, which can translate into contradictory findings. Notably, with increasing temperature, due to a decrease in functional oxygenated groups and an increase in aromatic structure, the material’s affinity to water is altered, the hydrophobicity of BC becomes higher than that of pristine biomass, and its capacity to retain water will be lower. Conversely, thanks to increased porosity, which changes the amount of water that can be adsorbed, BC produced at high temperatures can hold more water in its porous structure than BC prepared at lower ones [104].

The mechanical stability of biomass usually decreases during pyrolysis and correlates inversely with the porosity and directly to the density of the BC and temperature. The electric conductivity increases with higher thermal treatment, improving the graphitic carbons’ crystallinity and the carbon-packed domains’ density [105]. BC with high mechanical stability can be produced from feedstocks with high density and lignin content, making lignin, the constituent, more resilient to decomposition and the loss of structural complexity. Conversely, BC with higher grindability can be obtained by the torrefaction of biomass with a larger amount of hemicellulose (e.g., agricultural residues) compared to woody biomass. The decomposition of biomass to BC causes a reduction in its bulk density and an increase in its porosity and, therefore, a decrease in its thermal conductivity, depending on the pyrolysis temperature. Concerning the electric properties of BC, the reduction in oxygenated functional groups and the appearance of conjugated double bonds cause an increase in conductivity and electromagnetic shielding efficiency, which make BC suitable as an additive in various composite materials (e.g., building materials such as cement). Furthermore, the effectiveness of shielding against electromagnetic interference is enhanced concerning the pristine biomass.

### 2.3. Possible Biochar Applications

The various properties of BC reported above, including its high carbon content, larger surface area, well-developed porous structure, and a surface sufficiently enriched with functional groups, render it potentially pertinent for various applications. In Table 3, we have reported the current possible environmental BC applications.

BC production could be an alternative to mitigate climate change by carbon sequestration in soil, thus retaining half of the carbon fixed in biomass during photosynthesis and reducing CO_2_, NO_2,_ and CH_4_ emissions [73]. Mainly, BC shows long-term stability in soil. The mean carbon residence time in BC has been estimated to be around 90–1600 years, depending upon the labile and intermediate stable carbon components [73]. Due to these characteristics, BC can sequester carbon in soil, thus decreasing carbon dioxide emissions into the atmosphere and those of nitrous oxide and methane by biotic and abiotic mechanisms [73]. Experiments have demonstrated that the emission of greenhouse gases (including CH_4_ and N_2_O) can be avoided by pyrolyzing waste biomasses [107]. Concurrently, the pyrolysis process balances fossil fuel consumption by producing bioenergy.

Interestingly, BC has been estimated to be capable of tackling 12% of the current anthropogenic carbon emissions. Furthermore, thanks to its high carbon content, BC can work as a soil conditioner, mainly by improving the soil’s physicochemical and biological properties. BC increases soil water retention capacity by ~18%, reduces nutrient leaching [68], and neutralizes acidic soils, thereby enhancing plant productivity, seed germination, plant growth, and crop yields. Additionally, wet BC prevents soil desiccation [68]. While it has been reported that soils treated with BC demonstrated improved microbial population and activity [108], null or positive effects were observed in the earthworm population in soils amended with wood-based BC [109].

The production of BC itself is an economical and mutually beneficial strategy to manage and eliminate waste from animals and plants and reduce the pollution associated with it [108]. Furthermore, when waste biomass that is derived mainly from animal manure and sewage sludge is pyrolyzed, the hazardous microbial population that is possibly present is killed, thus reducing its possible negative impact on the environment and humans. Unfortunately, toxic heavy metals from sewage sludge and municipal solid waste could persist in BC, which must be carefully checked and handled correctly before long-term soil application [110].

A remarkable potential use of BC, one that is still too little investigated and controversial, is the production of bioenergy as an alternative to fossil fuel that could lower carbon emissions. In this regard, while slow pyrolysis allows a lower yield of liquid fuel and more BC, fast pyrolysis provides more liquid fuel (bio-oil) and less BC [111]. Evidence has demonstrated that BC can be successfully applied in environmental remediation because it is capable of adsorbing both organic and inorganic contaminants, such as pesticides, herbicides, PAH, dyes, and antibiotics, as well as non-biodegradable metal ions, which are highly toxic to all living organisms [72,74]. BC can enhance the composting process by improving its physicochemical properties and microbial activities and promoting the decomposition of organic matter. Also, more investigations are needed to evaluate BC compost’s agricultural/environmental performance [69]. Table 4 summarizes some of the advantages and disadvantages associated with the production and use of BC.

As evidenced in Table 4, we benefit from additional advantages by producing BC from biomass, including waste biomass. The cost necessary to produce BC is six-fold lower than that of commercially available activated carbon (AC), which, unlike BC, is deprived of some properties of BC, such as its ion exchange capacity [112]. Generally, BC does not require further processing to be activated, and, thanks to its non-carbonized fraction and maintained oxygen-containing groups such as carboxyl, hydroxyl, and phenolic surface functional groups, BC is capable of adsorbing both organic as well as inorganic contaminants and of interacting with soil contaminants [72,74]. BCs produced from sewage sludge and manure have a high nutrient content for soils, thus enriching their quality [68]. However, apart from the advantages of using BC, there is a series of possible fallouts, as reported, that need consideration. Among these, the long-term removal of crop residues, like stems, leaves, and seed pods, for producing BC could reduce the overall soil health by diminishing the number of soil microorganisms and disrupting internal nutrient cycling, with a possible negative impact on soil biota, including short-term adverse effects on earthworm population density. In this scenario, there is a dire need for further extensive research so that any possible issues associated with its usage can be effectively resolved.

#### 2.3.1. Xenobiotics Removal by Biochar (BC)

As reported in the previous section, BC is a porous material, and its porosity, depending on the production temperature, allows it to interact with water nutrients and other materials, including inorganic metal cations and organic pollutants. Due to its enhanced porous structure, surface area, functional groups, and mineral components, BC is an optimal absorbent material for solutions. Although BC that is produced through pyrolysis has a relatively moderate adsorption capacity (3.6–6.3 g/g for BC prepared at a temperature range of 300–700 °C) [113], this can be enhanced by modifying its physicochemical properties through acid, alkali, or oxidizing treatments, while the surface area can be altered mainly using acid treatments [114,115,116]. As an adsorbent, BC can absorb organic and inorganic contaminants, such as PAH and phthalate acid esters, and its help in improving the treatment of sewage wastewater containing organic xenobiotics has been widely reported [117]. In this context, there are several main mechanisms used by BC for capturing inorganic or organic pollutants, which have been included and are discussed in Table 5.

Interestingly, BCs produced at higher temperatures exhibited higher sorption efficiency for the remediation of organic and metallic contaminants in soil and water. Additionally, it is worth mentioning that the sorption of organic xenobiotics by BC is more favorable than that of inorganic ones. Concerning complexation with metal cations, the smaller the ionic radius of metals, the greater the adsorption capacity by BC.

#### 2.3.2. Not Only Adsorption

It is commonly reported that the principal mechanism by which BC removes toxic heavy metals and other contaminants, including organic pollutants, is adsorption. Its adsorptive efficiency mainly depends on the type and number of functional groups, surface area, CEC, etc. However, previous research studies and reviews on BC have evidenced the presence, either on the surface or inside its particles, of free radicals known as persistent free radicals (PFRs), the nature of which depends strongly on the pyrolysis conditions and the formation and characteristics of which mainly differ based on the feedstock types. In this regard, several recent studies have mainly focused on the role of BC-related PFRs in the degradation of organic xenobiotics, in addition to their adsorptive capacity. Odinga et al., in their recent work, reviewed the application of BC-derived PFRs in environmental pollution remediation [27], while Fang et al. investigated the reactivity of PFRs in BC and their catalytic ability to activate persulfate to degrade pollutants [28]. However, Odinga et al. also considered and commented on the possible environmental risks of PFRs from BCs, which represent the shadows associated with these chemicals and need further study, knowledge, and regulation before their extensive application [27].

## 3. Biochar-Derived Free Radicals

As previously mentioned, BC has a broad-prospective use in the treatment of environmental xenobiotics, in soil amendment, in photocatalytic and photothermal systems, for photothermal conversion, as electrical and thermal devices, and as 3D solar vapor-generation devices for water desalination [118,119,120,121]. All these potential uses are due to its high surface area and rich pore structure, which provide great physical absorptivity. They also depend on the chemical characteristics of BC, including the presence of PFRs [122,123]. In this regard, it is of paramount importance to clarify the formation mechanism of free radicals in BC for the optimal management of their properties and their more efficient and safer utilization [124].

### 3.1. Persistent Free Radicals (PFRs)

**Figure 6:**
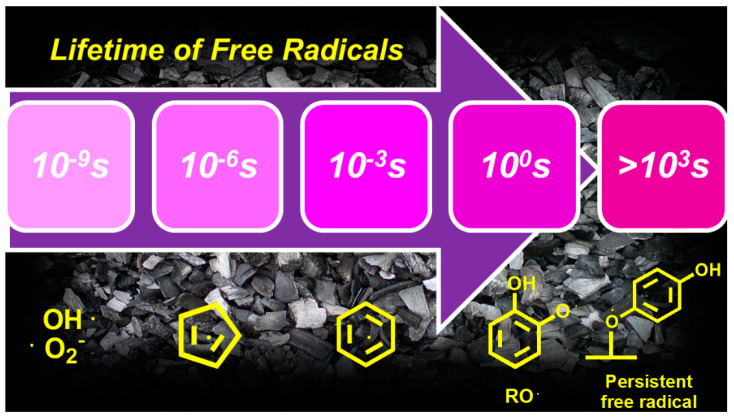
The lifetimes of different free radicals.

An atom or molecule with at least a lone pair of electrons is a chemical species characterized by significant instability and high chemical activity and is referred to as a free radical species [107]. Usually, free radicals are highly unstable and rapidly react with each other, thus being destroyed as soon as they form, with a consequent very short half-life. However, it has been found that in BC, some free radicals, named persistent free radicals (PFRs), like the radicals that naturally occur in the environment, known as environmental persistent free radicals (EPFRs), can remain stable for months and play a crucial role in the subsequent reactions of oxidative degradation carried out by BC containing them [25,107,125,126] (Figure 6).

**Figure 7:**
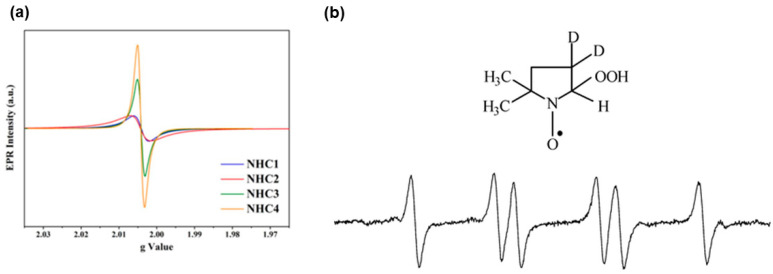
EPR analysis of PFRs generated on a N-doped hydro char prepared in a tube furnace heated to 600 °C for 1 h under N_2_ atmosphere (**a**) [127]. The image has been reproduced with permission from Elsevier under license number 5754160784661, received on 22 March 2024; EPR spectrum of the unstable free radical superoxide (O_2_•^−^) when trapped by DMPO to form a long-lived nitroxide (DMPO-OOH) (**b**).

Unlike other free radicals, PFRs are resonance-stabilized since they are bound to the external or internal surface of solid particles of BC. They can be analyzed by electron paramagnetic resonance spectroscopy (EPR) [25]. Figure 7a provides an example of an EPR analysis of the PFRs present on a solid N-doped hydro char prepared in a tube furnace at a temperature of up to 600 °C for 1 h under a N_2_ atmosphere [127].

**Figure 8:**
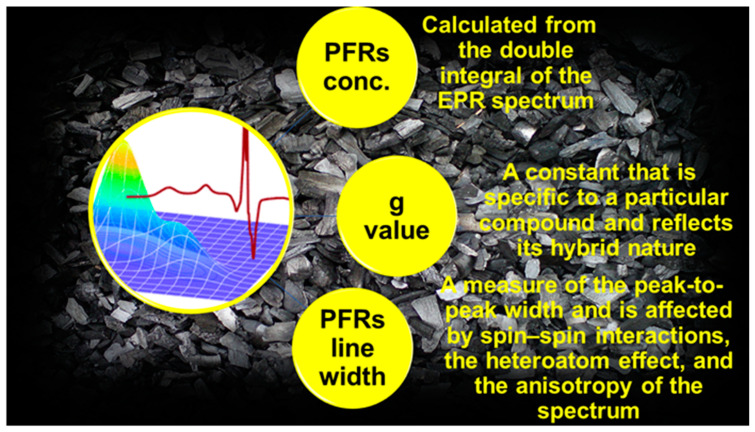
Information derived from EPR analyses.

Their lifetime under a vacuum appears infinite, while they react with molecular oxygen in the air, resulting in decay with time and the simultaneous production of reactive oxygen species (ROS). In this regard, PFRs act as transition metals like Fe^2+^, stimulating ROS production in aqueous systems. Unlike PFRs, ROS are detectable by EPR only when captured by a proper radical scavenger, such as 5,5-dimethyl-1-pyrroline-N-oxide (DMPO). Figure 7b provides an example of the EPR spectrum of the unstable free radical superoxide (O_2_•^−^) when trapped by DMPO to form a long-lived nitroxide radical (DMPO-OOH). In BC analyzed using the EPR technique, PFRs were previously detected in combustion-generated particulate matter (PM), sediments, and soils. PFRs can be categorized into three classes, i.e., oxygen-centered PFRs (OCPFRs), carbon-centered PFRs (CCPFRs), and oxygenated carbon-centered radicals (CCPFRs-O). The EPR analyses provide three parameters: the PFR concentration, the g-value, and the line width [107] (Figure 8).

PFR concentration is calculated from the double integral of the EPR spectrum and can reflect the content of PFRs in BC [128]. The g-value of PFRs is a constant specific to a particular compound, reflects its hybrid nature, and provides information about the type of radical [129]. The PFR line width in the EPR spectrum measures the peak-to-peak width. This is affected by spin–spin interactions (including electron–proton interaction and electron–electron interaction), the heteroatom effect, and the anisotropy of the spectrum [107].

The line width reflects the relaxation time of spinning electrons [130]. It has been reported that the oxidation processes that can occur using BCs mainly depend on PFRs and these parameters [125,131,132]. These parameters are, in turn, affected significantly by pyrolysis conditions, biomass types, the elemental composition of pristine biomass, and the presence of external transition metals (Table 6).

Qin et al. [132] found that the PFR concentrations in the same BC that were obtained at different temperatures and those in different kinds of BC obtained at the same temperature were significantly different. Tao et al. [135], as well as Xiang [136] and Huang et al. [24], found that in BCs from different feedstocks, the PFR concentrations first increased with increasing temperature, reaching a maximum around 500–600 °C, and then decreased with a further increase in temperature. The relations between the feedstocks’ properties or the BCs’ composition with the PFR concentrations were also demonstrated [133], and non-lignocellulosic-biomass produced lower PFR concentrations than lignocellulosic-biomass under the same pyrolytic conditions, perhaps due to their lower H/C and O/C atomic ratios [134]. The types of PFRs that can be produced during pyrolysis change in the pyrolysis process and change along with a temperature rise, as reported in Table 6. One study speculated that the reduction in oxygen content during biomass pyrolysis may account for the progressive conversion of oxygen-centered radicals to carbon-centered radicals [134].

#### Mechanism Proposed for PFR Formation during Biomass Pyrolysis

**Figure 9:**
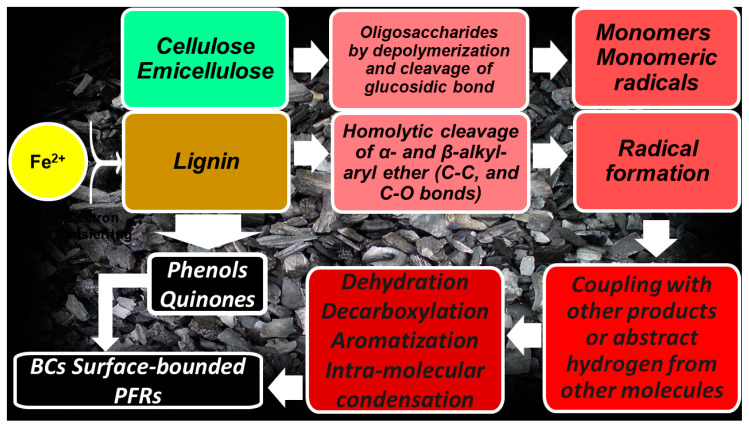
The PFR formation process.

**Scheme 1:**
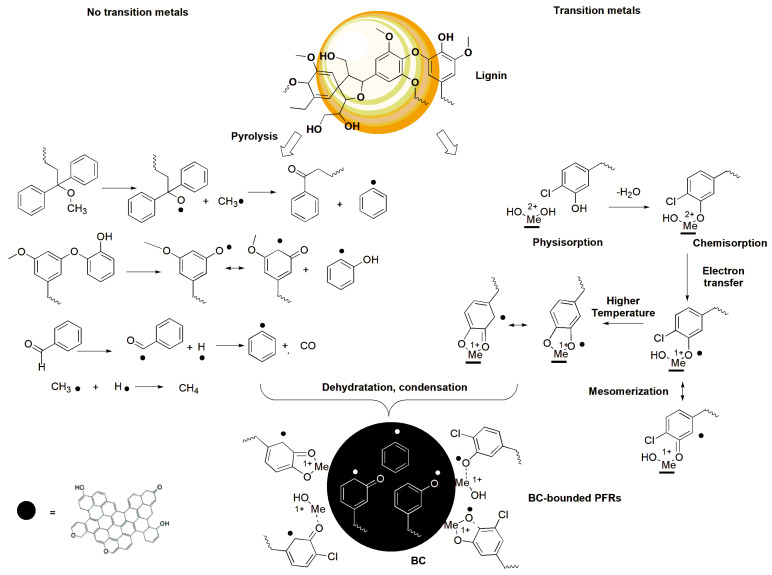
Possible mechanisms leading to the formation of BC-bounded PFRs from lignin. The orange sphere represents biomass, while the black sphere represents BC, the hypothetical structures of which, depending on pyrolysis condition, are shown at the bottom of the scheme.

**Scheme 2:**
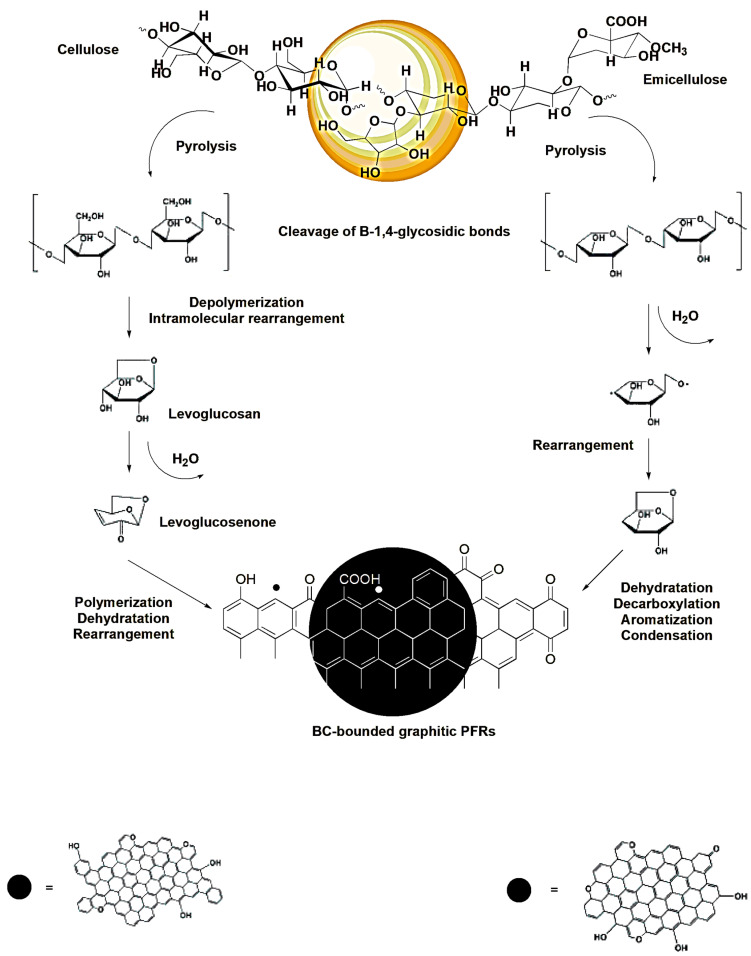
Possible mechanisms leading to the formation of BC-bounded graphitic PFRs from cellulose (**left side**) and hemicellulose (**right side**). The orange sphere represents biomass, while the black sphere represents BC, the hypothetic structures of which, depending on pyrolysis condition, are shown at the bottom of the scheme.

The several environmental sources of PFRs include atmospheric particulate matter (PM), contaminated soil, materials from thermal treatments of plastic and hazardous waste, tar balls, and the products deriving from the pyrolysis of biodiesel and biomass waste feedstocks at high temperatures [25]. Concerning BC-derived PFRs, it was observed that they mainly form in the post-flame and cool-zone regions of combustion systems and other thermal conversion processes. Although the actual mechanism by which PFRs form during pyrolysis remains not fully clarified, transition metals capable of electron transfer and the substituted aromatics molecules present in lignin have been recognized as critical factors of PFR formation. However, high concentrations of PFRs have also been detected in the product of combustion of non-aromatic cellulose in the absence of transition metals [135]. Based on the temperature of pyrolysis processes, during the production of BC, highly heterogeneous composite structures occur, comprising both labile and recalcitrant organic molecules, such as PAH, furans, and dioxins, as well as inorganic fractions including oxides, cations, anions, and free radicals [137]. These fractions, products of the incomplete combustion of biomass, may gradually form PFRs by different pathways, including or not including transition metals. The formed PFRs could be either only surface-stabilized or be surface-stabilized in metal-radical complexes [27]. Generally, the breaking of covalent bonds by heat, light, electricity, and chemical energy, is essential to form free radicals; during the pyrolysis process of lignocellulosic biomasses. Their main constituents, namely, cellulose, hemicellulose, and lignin, undergo different reaction pathways at various destructive pyrolysis temperatures of 300 °C, 300–400 °C, and 350–450 °C. Anyway, the presence of transition metals can strongly affect the possible formation of PFRs during pyrolysis. Figure 9 attempts to describe the possible series of events occurring during biomass pyrolysis that could lead to the formation of PFRs, which are also chemically described in Scheme 1 (concerning lignin) and Scheme 2 (concerning cellulose and hemicellulose).

First, the C-O and C-C covalent bonds of constituents of lignin are broken under heat, either via electron transfer by transition metals or not, to form free radical fragments, phenols, chinones, and other products of incomplete combustion. Simultaneously, the cleavage of the glucoside bonds of cellulose and hemicellulose that are present in biomass feedstocks occurs, causing depolymerization and the formation of other radicals. These first radicals can couple to form bio-oil, pyrolysis syngas (CO_2_, CO, CH_3_CH_3_, and CH_4_), and BC simultaneously or may abstract hydrogen from other molecules, forming further radicals [133,138]. Several chemical reactions can occur, including dehydration, decarboxylation with further emissions of CO_2_, CO, and H_2_O, aromatization, and intra-molecular condensation leading to the formation of the crystalline graphene structure and graphitic radicals. During pyrolysis, the elemental composition of biomass undergoes changes that cause mutations in the types of radicals that, upon entrapment onto the BCs’ surface and/or the formation of metal–radical complexes, form stable PFRs [24].

According to findings reported in the literature, the possible types of PFRs comprise (i) transition metal-mediated PFRs or (ii) PFRs forming inside organic matrices during biomass pyrolysis to give BC [139]. The transition metal-mediated PFR formation starts with the initial physisorption of an aromatic substituted molecule or of its degradation intermediate radicals, generated at 150–400 °C or under UV irradiation onto transition metal oxides such as ZnO, NiO, CuO, Fe_2_O_3_, and TiO_2_ or transition metal ions [140]. Then, chemisorption occurs by forming a chemical bond eliminating water or hydrogen chloride. Finally, a single electron is transferred from the substituted aromatics to the center of the transition metals, leading to the simultaneous reduction of metal and the formation of PFRs [140], the stability of which can be attributed to the synergy of metals and aromatic compounds [140]. A transition metal accepts an electron, and its valence changes from high to low during this process.

Unlike the PFRs discussed previously, PFRs formed inside the matrix of organic moieties are not related to the presence of transition metals [139]. Still, they are highly dependent on the relevant organic matter, while their concentration is significantly and positively correlated with the elemental carbon content [139]. In this case, PFRs are compared in terms of thermally treated particles, for which the breaking of chemical bonds in the precursor molecules during pyrolysis is the primary reason. At the initial pyrolysis stage, the homolytic cleavage of weak linkage bonds like the α- and β-alkyl aryl ether bonds, C-C, and C-O linkage resulted in the forming of free radicals in BC. The outer-surface free radicals would rapidly react and dissipate, resulting in a decrease in EPR signals. The free radical concentrations then increased with extended pyrolysis and during the cooling stage, thus accumulating many free radicals on the BC’s surface [139] and dramatically boosting the EPR signals. The free radicals formed in the matrix of the produced BC are probably protected from reacting with each other or other chemicals and are thus stabilized.

As mentioned earlier, the type of biomass and its elemental composition, the presence of oxygenated functional groups, the pyrolysis conditions (temperature, heating time, and heating rate), and the presence of external transition metal as well as phenolic compounds strongly affect both the concentration, structure, and type of PFRs present in BC. Notably, no radical is produced during the first stage of pyrolysis, providing the transition char (< 300 °C). Subsequently, in the second stage of pyrolysis (300–500 °C), amorphous char is produced, and oxygen-centered radicals and oxygenated carbon-centered radicals appear. In the third stage of pyrolysis at 500–700 °C, composite char is created, wherein the concentrations of PFRs, including carbon-centered and oxygenated carbon-centered radicals, drastically decrease. Finally, when turbostratic char is produced (> 700 °C), little or no PFRs are subsequently produced [27]. In the EPR, the g-factor values, even if they could change due to the presence of metal ions and temperature changes, are specific for a type of radical. Table 7 reports the main types of radicals recognizable in BC and their specific g-values.

### 3.2. PFRs: Light and Shadows

#### 3.2.1. PFR Light

It has been demonstrated that PFRs originating in BC by combustion in the presence or absence of external transition metals could play a vital role in several beneficial reactions, such as the PFR-mediated remediation and degradation of organic and inorganic pollutants by different actions and mechanisms, including oxidative and reductive processes (Table 8).

For instance, PFRs on BC can activate hydrogen peroxide (H_2_O_2_) or oxygen (O_2_), as well as persulfate (S_2_O_8_^2−^), to produce different free oxygenated radicals (ROS) that are capable of efficiently degrading organic contaminants such as chloro-biphenyl [32], phenolic compounds and polychlorinated biphenyls [21], diethyl phthalate [33], thiacloprid [123], and bisphenol A [25]. Moreover, organic chemicals can also be directly degraded on the BC surface by macromolecular free radicals without adding any radical activators [74]. The semiquinone-type radicals present in BC can oxidize As (III) [142]. At the same time, BCs can also exhibit the highly effective removal of Cr (VI) by reduction to Cr (III) using PFRs for industrial wastewater remediation [143,144,145,146,147].

**Figure 10:**
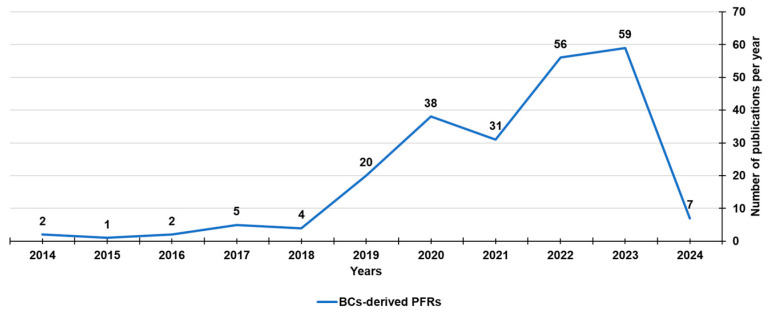
Number of publications on BC-derived PFRs from the year 2014 to the present, according to the Scopus dataset (reviews and chapters in the books included). The survey used the following keywords: persistent AND free AND radicals AND biochar.

Unfortunately, PFRs, by generating surface-bound hydroxyl radicals and free hydroxyl radicals in aqueous solution and also in the absence of H_2_O_2_, can induce various types of cardiovascular and pulmonary disease through ROS-induced oxidative stress (OS) [25]. PFRs and OH radicals that were detected in biological fluids generated ROS that induced an oxidant injury and modulated toxic responses in biological tissues [149]. Moreover, quinoid redox cycling is another possible path causing the formation of ROS from material containing semiquinone-type radicals, which could exert toxicity like that exercised by the combustion products present in cigarette smoke [150]. Although BC has beneficial effects on agricultural soil, the PFRs in BC could inhibit plant germination and growth when used in soil remediation. BC addition as a soil amendment has been reported to positively affect plant germination, growth, and yield [151,152]. In contrast, a negative impact has also been documented when BC-bounded PFRs induce ROS, which can inhibit seed germination and retard the growth of roots and shoots [32,35]. As shown in Figure 10, the formation and presence of PFRs in the BC produced by several biomasses have been widely documented and studied since 2014.

In this regard, in Table 9, we have reported a random selection of the main experimental works regarding the PFRs found in BCs, obtained by different biomasses conveyed in recent years (2019–2024). Table 9 also summarizes their reported applications, including mainly the oxidative degradation of organic environmental pollutants (51 papers), the removal of hazardous inorganic compounds from wastewater such as As (III) and Cr (VI) (12 papers), the degradation of biological samples, including bacteria (3 papers), hormones (4 works), genes of bacterial resistance (1 paper), and their use as electrical devices due to their electron and electron donor capacities (EAC and EDC).

**Figure 11:**
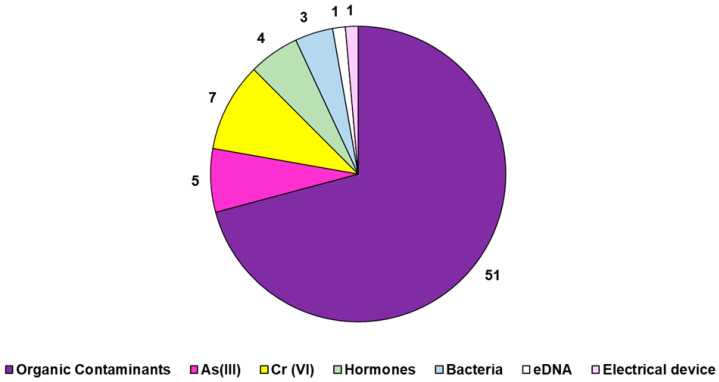
Number of publications for each type of EPR application found in the literature, considering a randomly selected population of 72 papers published from 2019 to 2023.

Figure 11 shows the relative abundances of the types of PFR applications concerning the 72 case studies considered here.

As for the mechanisms, many publications regarded the activation, which was sometimes photocatalytic, of PS, PMS, and PDS by BC. The employed BC was derived from different feedstock biomasses (bagasse powder, poplar and pine sawdust, cellulose, lignin, blue algae, waste straw, and other sources, as reported in Table 9), not doped, or doped with nitrogen atoms or different metals including Fe, Mn, Co, Ni, Zn. In these processes, the electron transfer promoted by metals and/or PFRs of a diverse nature, based on the pyrolysis conditions, generated ROS such as SO_4_•^−^, •OH, •O_2_^−^, •O_2_H and non-radical species (^1^O_2_), which carried out the oxidative degradation of different organic xenobiotics, including drugs, dyes, antibiotics, and hormones, as well as phenols or aromatic derivatives. Many other publications reported the use of BC to activate or photochemically activate O_2_ or H_2_O_2_ (Fenton-like systems) via metal and/or PFRs-mediated single-electron transfer. The generated ROS (•OH, •O_2_^−^, •O_2_H) and oxygen non-radical species (^1^O_2_) successfully oxidized several organic pollutants, degraded hormones, and eDNA and, in some cases, showed antibacterial effects against *E. coli* and *S. aureus*. Moreover, the capacity of BC to transfer electrons via transitional metals or PFRs was used to oxidize As (III) to As (V) or reduce Cr (VI) to Cr (III), thus resulting in helping to remove hazardous inorganic contaminants from industrial wastewater. A notable recent review reported on the efficiency of BC/layered double hydroxide composites as catalysts for the treatment of organic wastewater by advanced oxidation processes [213]. Several studies reported in this paper by Liu et al. demonstrated that degradation processes were based on radical reactions triggered by BC-associated PFRs [213].

#### 3.2.2. BC-Associated PFRs Shadows: Cytotoxicity and Biotoxicity

Despite the plethora of possible beneficial applications of BC, PFRs, as well as other free radicals and the toxic substances that compose BC, such as heavy metals, PAHs, dioxins, and perfluorochemicals (PFCs), are released into the environment during the pyrolysis process, thus representing a potential risk to the environment and humans [214]. Additionally, as well as other contaminants, the possible carbon allotropes formed during pyrolysis are severe contaminants in air, water, and soil [215]. Black carbon, carbon black (CB), carbon nanotubes, graphene, quantum dots, and fullerenes can possess distinct toxicity that depends on many factors, including the type of allotrope, particle size, form, structural defects, coating molecules, and grade of functionalization [215]. Understanding the toxicity of carbon nanomaterials and nanoproducts that are possibly present in BC is essential for human and environmental health, safety, and public acceptance. In this regard, recent studies have focused their attention on the adverse effects of BC due to its particle size and the various interactions with the environment that could occur [216,217]. Upon its application, BC may produce harmful environmental effects due to aging by oxidative or biological processes, leading to changes in its properties [218,219]. Additionally, higher toxicity has been reported for BC with micro- or nano-dimensioned particles. It has been reported that the presence of micro-BC (MBC) or nano-BC (NBC) can promote the release of heavy metal ions into the medium when applied to soil [214]. Kim et al. (2018) observed that BC particles with a particle size of less than 0.45 μm could increase the release and mobility of As in soil [220]. Regarding the biotoxicity of MBC/NBC, it has been previously reported that particle-induced oxidative stress is a crucial mechanism of MBC/NBC cytotoxicity, which increases as the particle size decreases. Also, the PFR concentration on the surface of particles with an aerodynamic diameter of less than 1 μm is the highest [139,221]. While several reviews and studies exist on the production and modification of BC, the reaction mechanisms, and the beneficial active role of BC in environmental remediation, the adverse effects and potential risks of BC have only recently been evidenced. The comprehensive phenomena and mechanisms involved in BC toxicity still require elucidation, especially in environmental media different from soil, including water and the atmosphere. It is imperative to systematically study and discuss the possible adverse environmental effects of BC application concerning various media, including water and the atmosphere, by determining the corresponding occurrence, detection, assessment, and avoidance measures. Worryingly, the current knowledge concerning the possible adverse effects on the environment and biota deriving from the extensive application of PFRs originating in BC is even more limited [222]. Although they are emerging as contaminants of increasing concern, their formation, fate, toxicity, and health risks are poorly known [222]. Thermal treatment, a common remediation technique to clean industrial soils, induces the formation of PFRs; this could paradoxically increase soil toxicity, which is contrary to the original remediation objective. For example, there is still little knowledge on the formation and toxicity of PFRs in soils contaminated by polycyclic aromatic hydrocarbons (PAHs) [223]. BC-derived PFRs, as well as those present in the environment and deriving from combustion and soil restoration, the burning of coal, wood, straw, cigarettes, oil, and other fuels, and from the restoration of organic contaminated soil, can enter the human body mainly through three pathways including the respiratory tract, from skin exposure, and via ingestion [149]. PFRs are not toxic to living beings and the environment, but they can stimulate the formation of other harmful substances and free radicals, including various types of ROS, when in the environment or in vivo [223]. As is well documented, ROS can interfere with the normal redox and metabolic processes, thus causing oxidative stress in biota [224]. Additionally, it has been reported that exposure to PFRs may induce cell degeneration or apoptosis and may affect the normal functions of the heart or lungs of humans [223]. So far, cytotoxicity and biotoxicity are the two categories of toxicity reported as attributable to PFRs (Table 10).

Usually, toxicity tests are carried out in research laboratories using both environmental samples and lab-prepared PFRs, such as those generated by MCP230 (a mixture of CuO and chlorophenol at 230 °C), DCB230 (a mixture of CuO, 0.2 μm amorphous silica and 1,2-dichlorobenzene at 230 °C), CGUFP (combustion-generated ultrafine particle) or other mixtures of transitional metals and substituted aromatic compounds. For cytotoxicity experiments, cultured cells extracted from the bronchial epithelium and rats are used, while biotoxicity essays are carried out on plants, fishes, rabbits, and worms. Generally, it was observed that exposure to PFRs causes oxidative stress. More specifically, the cytotoxicity tests evidenced cell variation with decreased numbers and activity, a disparity in protein expression, and DNA damage. Biotoxicity experiments revealed abnormalities in development and behavior, disease, and organ and tissue damage. Although BC can serve as an environmentally sustainable soil amendment material due to its ability to enhance several chemical properties of soil, such as pH, electrical conductivity, CEC, and organic carbon content, thus contributing to the overall improvement of nutrient retention in the soil, BC amendments with high concentrations of PFRs negatively affect crop growth.

Additionally, it has been found that PFRs used in aquicultural solutions inhibited the germination rate of different crops by ROS induction [149]. The oxidative stress brought about by the production of ROS can also damage the plasma membrane of the root system and hinder plant root growth. Moreover, PFRs induced neurotoxicity in *Cryptobacterium hidradenoma*, transforming it into a neurotoxin for soil organisms and thereby posing a threat to their survival.

## 4. Future Challenges and Risk Prevention Strategies

This review has evidenced that BC and mainly the PFRs generated during the pyrolysis processes performed to produce it could be double-edged weapons. BC is reported to be an eco-friendly and low-cost black gold with many beneficial properties, including the capability to remove organic and inorganic pollutants from water by adsorption processes and/or through its PFRs. However, several studies have reported that PFRs can be very dangerous to the environment and humans through a ROS-dependent mechanism. In addition to being produced by various common xenobiotics, PFRs can easily be converted into secondary pollutants, causing further biotoxicity. The still too-little-studied transport and transformation of PFRs in the environment can also affect the behavior of other substances, leading to potential environmental hazards that are not yet fully understood. Therefore, further exploration of the ecological impact of PFRs and the development of prevention and control measures are necessary. In this regard, although some progress has been made in terms of environmental risk and biotoxicity studies concerning PFRs, research is still in the initial stages, and there is an urgent need for systematic and in-depth studies on the production and transport of PFRs. A more in-depth understanding of the influence of environmental conditions on their occurrence is needed to control the external factors for reducing the negative output of PFRs and promoting their degradative action on xenobiotics. More rational knowledge about their toxicity mechanisms is necessary to have more precise toxicological equivalence regulation. A better strategy to prevent the risks associated with PFRs could be to avoid exposure to them by reducing contact with combustion sources, such as vehicle exhaust and cigarette smoke. Proper air filtration systems removing PFRs from indoor air and wearing protective masks or respirators could lower the possibility of contact with PFRs in outdoor environments. Additionally, treatments for limiting the adverse health effects associated with PFR exposure, such as using antioxidants, which can neutralize ROS, could be another strategy to protect humans from the adverse impacts of PFR exposure.

## 5. Conclusions and Future Perspectives

The pivotal role of BC-related PFRs in BC catalytic efficiency in water and soil pollution remediation has been reviewed in this paper. The main mechanisms by which PFRs can originate and the main methods to detect them on BC have been discussed. The key roles of feedstock biomasses and pyrolysis conditions in the formation of PFRs has also been reported. Several recent case studies concerning the critical role of PFRs in the catalytic oxidative degradation by BC of organic pollutants and in the removal of Cr/As and other metal ions in aqueous phase have been reported and discussed. It has been evidenced that for organic pollutants remediation, PFRs act as activator agents of oxidant substrates, which are subsequently involved in the degradation process. Otherwise, for metal ion removal, the PFRs on BC act as electron transfers for the adsorption and concomitant reduction/oxidation of metals by BC. Finally, PFRs on BC could also help mediate the recycling of Fe^3+^/Fe^2+^ in Fenton-like processes to enhance the efficiency of BC in pollutant removal. However, many aspects remain unclear concerning PFRs, including the influence of BC size on the evolution of PFRs, the exact relationship between the reactivity of BC and its size, and the overall roles and relative significance of PFRs, quinone moieties, and the carbon structure of BC in the activation of oxidizing agents and in the redox transformation of inorganic contaminants. Furthermore, while several studies about the laboratory applications of BC for wastewater remediation have been developed, future studies should concentrate on the up-scaled utilization of BC for the treatment of different real wastewaters, such as industrial wastewater and municipal wastewater. To this end, efforts are needed to design proper reactors and to develop methods for the large-scale production of the desired BC. Finally, to better contribute to the circular economy, the reutilization of spent BC should be given serious consideration.

## Figures and Tables

**Table 1:** Main BC production methods, temperature conditions, and yields.

	Temperature (°C)	Residence Time	Biochar (%)	Bio-Oil (%)	Syngas (%)	Refs
Pyrolysis	200–700	0.5–2 s	35	30	35	[86]
	500–1000	Hours/day	12	75	13	
HC	180–300	1–16 h	50–80	5–20	2–5	[87]
Gasification	750–900	10–20 s	10	5	85	[88]
Torrefaction	290	10–60 min	80	0	20	[89]
Flash carbonization	300–600	<30 min	37	--	--	[90]

HC = Hydrothermal carbonization.

**Table 2:** Main chemical and physical features of BCs.

Properties		Discussion
**Chemical properties**	Atomic ratio	⇓ O/C and H/C ratio for untreated biomass
	Elemental composition	⇑ Carbon content (> 95%) * ⇓ Hydrogen content (< 5%) * ⇓ Oxygen content (< 2%) *
	Energy content	⇑ Energy content with temperature (From 15–20 MJ/kg ^a^ to 30–35 MJ/kg ^b^ at 700 °C
	Fixed carbon (FC) ** Volatile matter (VM)	⇑ in FC (from 10% ^a^ to 90% ^b^ at 700 °C) ⇓ in VM (from 90% ^a^ to 10% ^b^ at 700 °C)
	Structural composition	Partially decomposed cellulose ^c^ Near totally decomposed hemicellulose ^c^ Partially decomposed lignin ^c^
		Release of O_2_ and H_2_ ⇓ Oxygenated functional groups in BC (OH and C=O groups) * ⇑ Highly stable aromatic structures in BC * (with maximum aromaticity at 500–800 °C) ⇑ Alkalinity and ability to neutralize acids in soils * ⇑ Unpaired negative charges that enable BC to accept protons
	pH value	⇑ pH value (from 5–7.5 ^a^ to 10–12 ^b^ at > 500 °C) ⇑ Ash
	Cation exchange capacity (CEC)	⇑ CEC for BCs produced at relatively ⇓ low temperatures
	Ash content (SiO_2_, CaO, K_2_O, P_2_O_5_, Al_2_O_3_, MgO)	⇑ With temperature
	Self-heating degradation during storage	⇓ Highly volatile content in BC ⇓ Risk of self-heating ⇑ Thermal stability ⇓ Risk of spontaneous combustion ⇓ Water content and microbial
**Physical properties**	Density and porosity	⇑ Weight-based energy density * at ⇑ temperature ⇓ Bulk density * (the volume-specific weight of a bulk material in a heap or pile) ⇑ Porosities at ⇑ temperature
	Surface area	⇑ Total surface area * (<800 °C) ⇓ Total surface area * (>800–1000 °C)
	Pore volume distribution Pore size distribution	⇑ Total pore volume * with ⇑ temperature Macropores (1000–0.05 μm) Mesopores (0.05–0.002 μm) Micropores (0.05–0.0001 μm (more than 80% of the total pore volume)
	Hydrophobicity Water-holding capacity (WHC)	⇑ Hydrophobicity ⇓ Affinity to water ⇑ Porosity and amount of water that can be absorbed
	Mechanical stability	⇓ Mechanical stability during carbonatization ⇓ Structural complexity during carbonization
	Grindability	⇑ Grindability compared to the parent material
	Thermal conductivity Heat capacity	⇓ Thermal conductivity in BC (from 1300 J/(kgK) ^a^ to 1000 J/(kgK) ^b^ at 500 °C)
	Electromagnetic properties	⇑ Conductivity ⇑ Electromagnetic shielding efficiency

* Depending on pyrolysis temperature: a higher degree of carbonatation at higher temperatures; ** after removing the volatile components, the carbon content that remains in the solid structure is called fixed carbon; ^a^ row biomass; ^b^ BC; ^c^ depending on biomass and pyrolysis temperatures involved (<650 °C decomposes almost all of the holocellulose (cellulose and hemicellulose); the temperatures required for decomposing lignin are several hundred degrees higher than those for holocellulose; CEC is defined as the number of exchangeable cations (e.g., Ca^2+^, Mg^2+^, K^+^, Na^+^, NH_4_^+^) that a material can capture, which directly depends on the surface structure and the presence of functional groups providing surface negative charges; ⇑ = high, higher, or an increase; ⇓ = low, lower or a decrease.

**Table 3:** Main possible applications of BC.

Application	Mechanisms	Refs.
Climate change mitigation	Sequestering carbon in soil ⇓ CO_2_ emissions into the atmosphere ⇓ NO_2_ emissions ⇓ CH_4_ emissions Tackling 12% of current anthropogenic carbon emissions	[73]
Soil improvement	⇑ Physicochemical and biological properties of soils ⇑ Water retention capacity of soil ⇓ Nutrient leaching ⇓ Acids in soils ⇑ Microbial population and microbial activity in soils Positive impacts on the earthworm population Preventing desiccation	[68]
Waste management	By pyrolyzing waste biomass *	[106]
Energy production	By conversion of waste biomass to BC by fast pyrolysis, thus providing liquid fuel (bio-oil)	[71]
Capturing contaminants	By adsorption of both organic pollutants and/or metal ions from soil and water	[72,74]
Composting	⇑ Physicochemical properties of composting ⇑ Composting microbial activities ⇑ Organic matter decomposition	[69]

* Including crop residues, forestry waste, animal manure, food processing waste, paper mill waste, municipal solid waste, and sewage sludge; ⇑ = high, higher, improved, or enhanced; ⇓ = low, lower, reduced, or decreased.

**Table 4:** Advantages and disadvantages associated with the production and use of BC.

Advantages	Disadvantages
Obviate to significant modification on Earth	Gaseous aerosol emissions during improper pyrolysis
Enhanced soil productivity	Environmental pollution from dust; erosion and leaching of BC particles
Higher food security	Ash could be at risk for respiratory diseases.
Solution of xenobiotics danger	BC can sequester water and nutrients not further available for crops
Addressing waste management	Not desired sorption of residual herbicides and pesticides
Reduced utilization of fossil fuels	Long-term removal of crop residues for producing BCs can reduce overall soil health by diminishing the number of soil microorganisms and disrupting internal nutrient cycling
Less expensive than activated carbon (AC)	Possible negative impact on soil biota
Improvement of living microbiology in soil	Short-term adverse effects on earthworm population density
Greater WHC than AC	No universal reduction in nitrous oxide emissions
Enhanced food web in soil	
Improved aeration in the soil	
Reduced loss of nutrients through leaching	

AC = Activated carbon; WHC = water-holding capacity.

**Table 5:** Main mechanisms by which BC can capture inorganic or organic contaminants.

Capturing Mechanism	Influencing Factors ^#^, Details °, Examples ^§^	Ref.
Sorption *	⇑ Surface area ^#^ Microporosity of BC ^#^ pH ^#^	[117]
Hydrogen bond formation **	For polar compounds °^,^**	
Electrostatic attraction/repulsion	For cationic compounds ° Interaction between positively charged cationic organic contaminants and negatively charged BC surfaces °^,^**	
Electrostatic outer sphere complexation	Due to metallic exchange with K^+^ and Na^+^ available in BC °^,^**	
Hydrophobic interactions ***	For non-polar compounds °	
Diffusion	Non-ionic compounds can diffuse into the non-carbonized and carbonized fractions of BC °	
Formation of surface complexes **	pH ^#^ Ionic radius ^#^ Between metal cations and -OH, -COOH on BCs °	
Precipitation	Lead precipitates as lead-phosphate-silicate in BC ^§^ Co-precipitates and inner-sphere complexes can form between metals and organic matter/mineral oxides of BC ^§^	

* From water/soil onto biochar; ** for BCs produced at relatively lower temperatures; *** for BCs produced at higher temperatures; ⇑ = high, higher, improved, enhanced; In the table, the voices with # are influencing factors, those with ° are details, and those with § are examples.

**Table 6:** Factors influencing PFR formation in BC.

Parameter	Influencing Factors	Specifications	Observations	Ref.
PFRs concentration	Biomass type	Cow manure, rice husk, others (< 500 °C)	≠ Concentrations	[132,133]
		Non-lignocellulosic biomass with ⇓ H/C and O/C	⇓ Concentration	[134]
		Lignocellulosic biomass	⇑ Concentration	
	Temperature	300 °C, 700 °C	≠ Concentrations	[132]
		Maximum concentration at 600 °C		[135]
		Maximum of concentration at 500–600 °C		[24,136]
	Transition metals	Adsorb onto biomass and transfers electrons from polymer to metal center during pyrolysis	⇑ Concentration	[32]
Type of PFRs	Temperature	200–300 °C	Oxygen centered radicals	[24]
		400 °C	A mixture of oxygen and carbon-centered radicals	
		500–700 °C	Exclusively carbon centered radicals	

≠ = Different; ⇓ = low, lower, reduced, decreased; ⇑ = high, higher, increased, improved.

**Table 7:** Features and g-values of the main PFRs forming in BC.

Radicals	g-Value	Features
Carbon-centered radicals	<2.003	Susceptible to oxidation in air
Carbon-centered radicals adjacent to an oxygen atom (oxygenated carbon-centered radicals)	2.003–2.004	Susceptible to oxidation in air
Oxygen-centered radicals	>2.004	More stable in an atmospheric environment
Semiquinone radicals (oxygen-centered)	>2.0045	More resistant to reacting with molecular oxygen in the ambient environment
Phenoxy radicals (oxygenated carbon-centered radicals)	2.0030–2.0040	Susceptible to oxidation in air
Cyclopentadienyls (carbon-centered radicals)	<2.003	Susceptible to reacting with molecular oxygen in the ambient environment

**Table 8:** Main actions and mechanisms by which PFRs remediate/degrade environmental xenobiotics.

EPFRs Actions	Degraded Substances *	Mechanism	Refs.
Activation of H_2_O_2_ by single electron transferring	SMX, CIP, SMT, TC, OG, MNZ, ERF benzene	Oxidation by the production of ROS (OH• #, HO_2_•, O_2_•^−^)	[32,141]
Activation of O_2_ by single electron transferring	Degradation of organic compounds Chloro-biphenyl Phenolic compounds Polychlorinated biphenyls Diethyl phthalate Thiacloprid Bisphenol A	Oxidation by the production of radical superoxide (O_2_•^−^)	[21,25,33,123]
Activation of persulfate (S_2_O_8_^2−^)	X-3B, SMT, CTC, SMX, TC, MB, SDZ, OG	Oxidation by the production of sulfate radicals (SO_4_•^−^)	[141]
Direct activity of macromolecular radicals on the BC surface	Direct degradation of organic chemicals	Oxidation	[74]
Direct activity of semiquinone-type radicals	As (III) removal	Oxidation	[142]
Direct activity of PFRs	Removal of Cr (VI)	Reduction to Cr (III)	[143,144,145,146,147]
Catalytic effects	Detoxification of environmental xenobiotics	Generation of activated species Stimulation of the microbial biotransformation	[74]
Ions’ exchange	Enhancement of agricultural soil performance	Maintenance of CEC in soils	[148]
Electron-hole pair formation	Photocatalytic degradation of contaminants under Vis irradiation	Electrons in free radicals can be transformed from the valence band to the conduction band under irradiation	[45]

* Degraded or removed; SMX = sulfamethoxazole; CIP = ciprofloxacin; SMT = sulfamethazine; TC = tetracycline; OG = orange; MNZ = metronidazole; # free or surface bond; KET = ketoprofen; CTC = chloro-tetracycline; SDZ = sulfadiazine; MB = methylene blue; ERF = enrofloxacin (photocatalytic degradation); X-3B = reactive brilliant red X-3B.

**Table 9:** BC-derived PFRs and their applications as described previously, reported in the years 2019–2024.

Biomass	Pyrolysis °C/Time	BC-Name	Active Radicals	Radical Mechanisms	Application ^1^ Degraded Compound ^2^	Refs.
Sawdust	700 °C/1 h	Fe^0^-BC-700	SO_4_•^−^ PFRs OH•	Activation of PMS by Fe^0^ Activation of PMS by PFRs	BPA ^2^	[153]
Waste wood	500 °C, 700 °C	Fe^0^-BC	SO_4_•^−^ PFRs OH•	Production of PFRs by Fe^0^ Activation of PS by Fe^0^ Activation of PS by PFRs	TDWW ^2^	[154]
Camellia seed husks	400 °C/2 h	OBC-Fe_3_O_4_	SO_4_•^−^ PFRs OH•	Activation of PS	TC ^2^	[155]
Maize straw	900 °C/2 h	NBC1	• O_2_^−^ SO_4_•^−^ PFRs OH•	Activation of PS	(86.6%) TC ^2^	[156]
Sawdust	300 °C, 700 °C	SBC	SO_4_•^−^ PFRs OH•	Activation of PS	AO-7 ^2^	[157]
N.R.	200 °C, 500 °C	N.R.	PFRs • O_2_^−^	UV-induced interaction PFRs/DOM and • O_2_^−^ production	RhB ^2^	[158]
Sewage sludge	500 °C/4 h	HNO_3_-BC	PFRs • O_2_^−^ •OH •O_2_H	Activation of H_2_O_2_	CIP ^2^	[35]
Wheat straw	500 °C/2 h	BC/Fe (III)	SO_4_•^−^ PFRs OH•	Activation of PS by PFRs	SMX ^2^	[159]
Sawdust	700 °C	BC700	SO_4_•^−^ PFRs OH•	Activation of PDS by PFRs	CA ^2^	[160]
Pine needle	500 °C/2 h	Fe/Mn/BC	•OH	Activation of H_2_O_2_ by Fe (II), Mn (II) and PFRs (FeMn/BC/H_2_O_2_ photo-Fenton system)	Naphthalene ^2^	[161]
Sewage sludge	500 °C/4 h	SS-BC	PFRs • O_2_^−^ •OH •O_2_H	Activation of O_2_ and H_2_O_2_ by PFRs Degradation of PNP by PFRs	CIP ^2^	[162]
Swine manure	600 °C	SBC	OCPFRs CCPFRs-O • OH •O_2_H	Activation of oxygenated species by OCPFRs and CCPFRs-O (heterogeneous Fenton-like systems SBC/H_2_O_2_)	SMT ^2^	[163]
Wheat straw	300 °C, 600 °C	BC300 BC600	•OH •O_2_H	Goethite (Gt)-mediated activation of H_2_O_2_ (Fenton-like system)	OFX ^2^	[164]
Wheat straw	500 °C/2 h, 800 °C/2 h	CoBCX	SO_4_•^−^ PFRs OH•	Cobalt and PFR-mediated activation of PMS via O_2_	ATZ ^2^	[165]
Various crop straws	450 °C, 550 °C 650 °C	BC450,550 BC650	SO_4_•^−^ • O_2_^−^ OH•	BC-mediated activation of PS by electron transfer	SDZ ^2^	[166]
Tobacco steam	300℃, 500℃ 700℃	T-BC	ROS	OCPFR-mediated activation of O_2_ in the water	PNP ^2^	[167]
Pruning wastes of apple trees	400 °C, 550 °C 700 °C	BC400, BC550 BC700	SO_4_•^−^ PFRs	BC and PFR-mediated activation of PS	ACT ^2^	[168]
Camphor leaves	400 °C/6 h	Fe (TPFPP)/BC	SO_4_•^−^ PFRs OH•	PFRs-mediated electrons transferring to iron porphyrin-loaded BC ^3^	PFOA ^2^	[169]
Corn stalks	240 °C/4 h	hydrochar	•OH	Electrode and PFR-mediated generation of ROS	2,4-DCP ^2^	[170]
Wheat straw	450 °C/4 h	Co3O4-BC	SO_4_•^−^ PFRs OH•	Co_3_O_4_-BCmediated activation of PMS	CAP ^2^ FF ^2^ TAP ^2^	[171]
Wheat straw Urea Iron salts	800 °C/1 h	Fe-N-BC	SO_4_•^−^ PFRs •OH • O_2_^−^	Fe, N co-doped BC and PFR-mediated activation of O_2_ and PS	AO7 ^2^	[172]
*Candida utilis*	700 °C/2 h	NCS-x	SO_4_•^−^ PFRs OH•	Activation of PMS by nitrogen-doped biochar nanosheets (NCS-x) using molten salt (NaCl and KCl) in the pyrolysis process	BPA ^2^ BPF ^2^ BPS ^2^ BPAF ^2^	[173]
Pine needles	500 °C	nFe3O4/BC	PFRs •O_2_H •OH • O_2_^−^	Activation of H_2_O_2_ by nano-magnetite supported biochar via Fe (III)/Fe (II) cycling and electron transfer with the PFRs	Ethylbenzene ^2^	[174]
Sewage sludge	800 °C/3 h	SM-(0.5:1)	SO_4_•^−^ PFRs OH•	Activation of PMS by nitrogen-doped sludge biochar with different ratios of melamine in acidic	Cationic/anionic dyes ^2^	[175]
Elephant grass	350 °C, 600 °C 900 °C 30–120 min	EG	OCPFRs	OCPFR-mediated oxidation	CV ^2^	[176]
Sunflower-straw	N.R.	SSBC	SO_4_•^−^ PFRs OH•	Enhanced Fe (II) activation of PS via BC and PFRs	Benzoic acid ^2^	[177]
Pine chips	500 °C	OP5 RP5	SO_4_•^−^ PFRs •OH • O_2_^−^ •O_2_H	EDC-involved structures, Fe^3+^ and BC (PFR)-mediated activation of PS in a Fenton-like reaction system using H_2_O_2_ and NaBH4	2,4-DCP ^2^	[178]
Rice straw	350 °C, 500 °C 700 °C	BCs MBCs BDOMs	PFRs •OH	Direct photocatalytic degradation in BCs and MBCs solutions by Xenon-lamp Oxygen reduction by FPRs of BCs and MBCs BDOM-mediated generation of ROS	SMX ^2^ CAP ^2^	[179]
Pomelo peels	600 °C	Fe@PP-Hy-Py	PFRs •OH • O_2_^−^	Amorphous Fe (0)-mediated formation of PFRs Fe (0)-mediated reduction of PNP EPFR-mediated oxidation of PNP via ROS (O_2_ and H_2_O_2_) activation	PNP ^2^	[180]
Softwood pine	823–873 K	US-BC BC-P BC-P-DEA US-BC-P-DEA US-BC-P-DEA	PFRs •OH • O_2_^−^ •O_2_H	Reinforcement of PFRs concentration doping BCs with Ni and Pb Activation of H_2_O_2_ by PFRs	Phenol ^2^	[181]
Camphor leaves	500 °C/1 h	Fe (VI)/BC-2	Fe(Ⅴ)/Fe(Ⅳ) PFRs •OH	Fe (VI)-BC (PFRs)-mediated electron transferring and generation of ROS	AZT ^2^	[182]
Bagasse powder	800 °C	DBC800 PBC800-A	SO_4_•^−^ PFRs •OH• O_2_^−^ •O_2_H	Enhanced BC-mediated activation of PS Improved PFR generation by natural endogenous minerals	TC ^2^	[183]
*Eichhornia* *crassipes* Iron salts	400 °C/2 h	MBC	PFRs •OH• O_2_^−^•O_2_H	Fe (II)-BC-mediated activation of H_2_O_2_ (Fenton-like system)	MNZ ^2^	[184]
Poplar and pine sawdust	300–500 °C	PO xxx PI xxx	SO_4_•^−^ PFRs •OH• O_2_^−^	Activation of PMS by CCEPFRs-O and CCEPFRs in BC	TC ^2^ CTC ^2^ DOX ^2^	[185]
*S. alfredii*	Air-dried	Metal@P	•O_2_H	PFR generation by the thermochemical behaviour of Mn and Zn Electron transfer Activation of PDS by PFRs in Fe/Zn@PB9/PDS system AOPs	Imidacloprid ^2^	[186]
Sludge	N.R.	N.R.	SO_4_•^−^ PFRs •OH• O_2_^−^	Production of ROS via PFRs Mn-mediated electron transfer through Mn-doped sludge-based biochar (BC) after mediation	CIP ^2^	[187]
Cellulose Lignin	200–1000 °C	C200, C500 C1000 L200, L500 L1000	SO_4_•^−^ PFRs • O_2_^−^	Activation of PS adsorbed onto BCs via PFRs, oxygen-containing functional groups, and defective structures of BCs	OFX ^2^	[188]
Chestnut shell KMnO_4_	700 °C/1 h 400 °C/1 h	Mn-BC	PFRs	Mn-improved electron-transfer	OTC ^2^	[189]
Spent coffee TiO_2_	300 °C 500 °C 600 °C	SBC500	PFRs •OH• O_2_^−^•O_2_H	Activation of H_2_O_2_ by Ti-doped H_2_SO_4_-modified biochar (SBCs) (Photo-Fenton-like system)	MO ^2^	[190]
RS	550 °C/2 h	BC-α-Fe2O3/MgO	PFRs •OH• O_2_^−^•O_2_H	UV light activation of PFRs Production of O_2_ upon NPA degradation O_2_ activation by PFRs	NPA ^2^	[191]
Sewage sludge	400 °C/2 h	SDBC	PFRs •OH• O_2_^−^•O_2_H	O_2_ activation by PFRs promoted by HNO_3_ or NaOH environmental	*p*-Chlorophenol ^2^	[192]
Peanut hull	700 °C/2 h	BC-Fe-1-Zn	SO_4_•^−^ PFRs •OH	Activation of PS by bimetal-modified peanut hull-derived biochar via Fe and Zn oxides and oxygen-containing functional groups active sites	TC ^2^	[193]
Blue algae	700 °C	Z-700 FeOX@BC	SO_4_•^−^ PFRs •OH •O_2_^−^•O_2_H	• O_2_^−^ production by FeOX (zero-valent iron and iron oxide) C=C, C=O, O-C=O, Fe-O functional groups and PFRs promoted the activation of PDS	TC ^2^	[194]
Biomass	300–1000 °C	N.R.	SO_4_•^−^ PFRs •OH • O_2_^−^	Activation of PS and PMS by physically and chemically modified BCs using acid/alkali treatment and metal doping via PFRs	PPCPs ^2^	[195]
Chicken feathers	350 °C/4 h 800 °C/4 h	MBC35@FH MBC80@FH	SO_4_•^−^ PFRs •OH • O_2_^−^	Activation of PDS by the transformation of Fe species, oxygen-containing functional groups, pyrrolic nitrogen, and PFRs to produce ROS	TPhP ^2^	[196]
Pine needles	300–900 °C	BC300-900	SO_4_•^−^ PFRs •OH • O_2_^−^	Activation of PMS by BC via ROS production or electron transfer capability	OFX ^2^ ENR ^2^ FLE ^2^	[197]
PolyS	220 °C/2 h ^$^ 500 °C /2 h ^#^ 900 °C /2 h ^#^	BC500 + PS BC900 + PS BC500 BC900	SO_4_•^−^ PFRs •OH • O_2_^−^	Activation of PMS using CCEPFRs on BC aged by microbial fermentation for ROS production	SDZ ^2^ OFX ^2^ DOX ^2^	[198]
Red mud Wheat crop	700 °C /2 h	MRBC	SO_4_•^−^ PFRs • OH	PDS activation by the active sites of MRBC, such as Fe (II) and PFRs	LFX ^2^	[199]
Various sludges	300–900 °C 2 h	S-HPBC S-PBC S-HBC	SO_4_•^−^ PFRs •OH • O_2_^−^	Activation of PS by PFR-mediated electrons transferring activity Electrons transferring to Cr (VI) by PFRs	TC ^2^ Cr (VI) ⇒ Cr (III) ^1^	[200]
Peanut shells	500 °C/4 h	BC-Ce	OFGs, CCPFRs	Electrons transferring to Cr (VI) by OFGs, CCPFRs; oxygen vacancies and graphitic structure in BC-Ce promoted by CeO_2_	Cr (VI) ⇒ Cr (III) ^1^	[201]
Rice husk	400 °C/1 h	BC400	OH• H_2_O_2_	(pH acid) Activation of O_2_ by phenol OH and semiquinone types of PFRs	As (III) ⇒ As (V) ^1^	[142]
			Semiquinone-type PFRs Quinoid C=O H_2_O_2_	(pH alkaline) Activation of O_2_ by phenol OH and semiquinone types of PFRs		
Rice husk	550 °C	RH-BC	PRFs	Promotion of OCPFRs by BC-inducted Cr (VI) degradation	Cr (VI) ⇒ Cr (III) ^1^	[143]
Stalk	450 °C/90 min	NBC	PFRs	N-doped BC-mediated evolution of PFRs for the transformation of Cr (VI)	Cr (VI) ⇒ Cr (III) ^1^	[144]
Rice husk	500 °C/2h	MGBs	PFRs •OH • O_2_^−^	Efficient surface Fe (III)/Fe (II) cycling via electron transfer with the PFRs of magnetic greigite/BC composites (MGBs)	Cr (VI) ⇒ Cr (III) ^1^	[145]
Sludge	220 °C/2h	BC	OCPFRs	UV-Vis photo-irradiation enhanced the production of PFRs Action of OCPFRs as electron donors to transform Cr (VI) into Cr (III)	Cr (VI) ⇒ Cr (III) ^1^	[146]
Sludge	120 °C	SBC120	OCPFRs	OCPFR-mediated electrons transferring to Cr (VI) in neutral solutions	Cr (VI) ⇒ Cr (III) ^1^	[147]
	270 °C	SBC270	CCPFRs	CCPFR-mediated electrons transferring to Cr (VI) in neutral solutions		
Rice husk	400 °C/1 h	rUBC, rDBC	Quinoid C=O PFRs	Quinoid C=O and PFR-mediated oxidation of As (III)	As (III) ⇒ As (V) ^1^	[202]
Maize straw powder	500 °C/2 h	FhBC	PFRs • O_2_^−^ •OH	Fe and PFR-mediated activation of O^2^ and H_2_O_2_	As (III) ⇒ As (V) ^1^	[203]
Sewage sludge	270 °C/2 h	SBC	SO_4_•^−^ PFRs •OH • O_2_^−^	Activation of PS by SBC via PFR-mediated electrons transferring	As (III) ⇒ As (V) ^1^	[204]
Pinewood	600 °C/1 h	Fe/HC	• O_2_^−^ •OH	Activation of O_2_ and H_2_O_2_ by CCPFRs	Estrogens ^2^	[205]
Rice straw	500 °C/1 h	BiPB	•OH PFRs	Generation of •OH by Bi/Bi_2_O_3_ and PFRs	Estrone ^2,^*	[206]
Anaerobic digestion sludge	400 °C 600 °C 800 °C 1000 °C	ADSBC 400 ADSBC 600 ADSBC 800 ADSBC 1000	SO_4_•^−^ PFRs OH•	BC-mediated activation of PDS	Dyes ^2^ Estrogens ^2^ Sulfonamides ^2^ *E. coli* ^2^ Others ^2^	[207]
Walnut shell	700 °C/1 h	BC700	PFRs	Oxidation by PFRs-mediated electron transfer	E1 ^2^ E2 ^2^ E3 ^2^	[208]
*Caragana* *korshinskii*	650 °C/3 h	ACB-K-gC3N	PFRs h^+^•OH• O_2_^−^	Electron photogeneration and PFR-mediated H_2_O and O_2_ activation	*S. aureus* ^2^ *E. coli* ^2^ RhB ^2^ TA ^2^ NOR ^2^ CAP ^2^	[209]
Pinewood	600 °C	Ag^0^-PBC	PFRs •OH • O_2_^−^	UV-light-promoted excitation of the electron-hole pairs and, subsequently, the production of ROS Enhanced ROS generation by PFRs	MB ^2^ *E. coli* ^2^	[210]
Rice straw	400 °C, 700 °C 120 min	Nano-BC	PFRs •OH • O_2_^−^	Oxidation and damage by ROS	eDNA ^2^	[211]
Rice straw	500 °C	RS-BC	Quinones Phenols PFRs	By electron acceptor capacity (EAC) By electron donor capacity (EDC)	⇆ Redox property ^1^ Electronic storage ^1^	[212]

In the table, the voices with ^1^ are applications, while those with ^2^ are degraded compounds; BCs = biochar; MBCs = modified-biochar; BDOMs = biochar-derived dissolved organic matters; PMS = peroxy-mono-sulfate; BPA = bisphenol A; PS = persulfate; TDWW = textile dyeing wastewater; TC = tetracycline; (TDWW); SBC = sawdust biochar; AO-7 = acid orange 7; RhB = rhodamine B; DOM = dissolved organic matter in BC; CIP = ciprofloxacin; SMX = sulfamethoxazole; PDS = peroxydisulfate; CA = clofibric acid; WW = wastewater; ⇆ = reversible; PNP = p-nitrophenol (water pollutant); SMT = sulfamethazine; SDZ = sulfadiazine; OCPFRs = oxygen-centered environmental persistent free radicals; CCPFRs-O = carbon-centered environmental persistent free radicals with oxygen atoms; * photocatalytic; CCPFRs = carbon-centered environmental persistent free radicals; BiPB = bismuth-containing BC; PFX = pefloxacin; OTC = oxytetracycline; CTC = chlorotetracycline; OFX = ofloxacin; AZT = atrazine; TMP = trimethoprim; AOPs = advanced oxidation processes; ACT = acetaminophen; PFOA = perfluorooctanoic acid; 3 degradation efficiency in presence of ascorbic acid (AA); 2,4-DCP = 2,4-dichlorophenol; CAP = chloramphenicol; FF = florfenicol; TAP = thiamphenicol; CV = crystal violet dye; MB = methylene blue; MNZ = metronidazole; DOX = doxorubicin; xxx = refers to the temperature of pyrolysis process; NPA = N-phosphono methyl iminodiacetic acid (organophosphorus pesticide (OP); NOR = norfloxacin; E1 = estrone; E2 = 17-estradiol; E3 = estriol; PPCPs = pharmaceuticals and personal care products; TPhP = triphenyl phosphate; ENR = enrofloxacin; FLE = fleroxacin (FLE); ^$^ HTC = hydro-thermal carbonization; ^#^ HTP = high temperature pyrolysis; PolyS = polystyrene; OFGs = oxygen-containing functional group; S-HPBC = S-doped hydrothermal + pyrocarbon BC; S-HBC = S-doped hydrochar, S-PBC = S-doped pyrocarbon.

**Table 10:** Potential toxic hazards caused by PFR exposure.

Target	Danger	Material Source	Refs.
Cells	⇑ Lungs’ T (Th1, Th2, Th17) cells	PM, DCB230, MCP230	[225,226]
	⇓ P450 activity	PM, MCP230	[227]
	Cardiomyocytes’ apoptosis	DCB230	[228]
	⇓ Survival of gastric epithelial cells	BaP–Na montmorillonite	[229]
	Loss of normal morphology of pulmonary epithelial cells	DCB230	[230]
	Mitochondrial depolarization	DCB230	[226]
	Changes in VEGF	ZnO/MCB	[231]
Enzymes Proteins Genes	Altered expression activity of Cyp1a, Cyp2b, Cyp2e1, Cyp2d2, Cyp3a and other genes	DCB230, MCP230	[225]
	⇑ Expression levels of peroxiredoxin-6 Cofilin 1, annexin A8	MCP230, CGUFP, ZnO/MCB	[226]
	⇓ of GSH, GPx, SOD	ZnO/MCB	[232]
Organs and tissues	Altered normal renal hemodynamics and urodynamics	N.R.	[233]
	Liver damage	N.R.	[234]
	Impairment of left ventricular function	DCB230	[235]
	Airway hyperresponsiveness Lung inflammation	MCP230	[236]
Individuals	Abnormalities in zebrafish	DCB230	[237]
	⇓ Growth and reproduction of luminescent bacteria	PM	[238]
	Altered behavior of *Caenorhabditis elegans*	Biochar	[239]
	⇓ Energy consumption	MCP230	[240]
Disease	⇑ Severity of the flu	DCB230	[241]
	Asthma	MCP230	[226]
	Cardiovascular disease and dysfunction	DCB230	[228]
Other damage	Oxidative stress	DCB230, ZnO/MCB	[232]
	DNA damage	BaP	[46]
	Lipid peroxidation	MCP230	[242]

⇑ Improved, increased; ⇓ decreased, inhibited; PM = particulate matter; DCP230 = 1,2-dichlorobenzene at 230 °C; MCP230 = mono-chlorophenol at 230 °C; MCB = mono-chlorobenzene; CGUFP = combustion generated ultrafine particle; N.R. = not reported; GSH = glutathione; GPx = glutathione peroxidase; SOD = superoxide dismutase; VEGF = vascular endothelial growth factor.

## Data Availability

Not applicable.

## Supplementary Materials

The following supporting information can be downloaded at: https://www.mdpi.com/article/10.3390/toxics12040245/s1, Table S1. Main sources and general application of BCs; Table S2. Fast and slow pyrolysis details; Table S3. Techniques typically used to characterize BCs in terms of their physicochemical, surface, and structural characterization; Table S4. Properties of BCs produced from various feedstocks at various production temperatures.

## References

[1] Zhang R., Zhang R., Zimmerman A.R., Wang H., Gao B. (2023). Applications, Impacts, and Management of Biochar Persistent Free Radicals: A Review. Environ. Pollut..

[2] Wang J., Wang S. (2019). Preparation, Modification, and Environmental Application of Biochar: A Review. J. Clean. Prod..

[3] Xiong X., Yu I.K.M., Cao L., Tsang D.C.W., Zhang S., Ok Y.S. (2017). A Review of Biochar-Based Catalysts for Chemical Synthesis, Biofuel Production, and Pollution Control. Bioresour. Technol..

[4] Behera B., Selvam S.M., Dey B., Balasubramanian P. (2020). Algal Biodiesel Production with Engineered Biochar as a Heterogeneous Solid Acid Catalyst. Bioresour. Technol..

[5] Lee J., Kim K.-H., Kwon E.E. (2017). Biochar as a Catalyst. Renew. Sustain. Energy Rev..

[6] Zhang L., Lim E.Y., Loh K.-C., Ok Y.S., Lee J.T.E., Shen Y., Wang C.-H., Dai Y., Tong Y.W. (2020). Biochar Enhanced Thermophilic Anaerobic Digestion of Food Waste: Focusing on Biochar Particle Size, Microbial Community Analysis and Pilot-Scale Application. Energy Convers. Manag..

[7] Supraja K.V., Kachroo H., Viswanathan G., Verma V.K., Behera B., Doddapaneni T.R.K.C., Kaushal P., Ahammad S.Z., Singh V., Awasthi M.K. (2023). Biochar Production and Its Environmental Applications: Recent Developments and Machine Learning Insights. Bioresour. Technol..

[8] Awasthi M.K., Wang M., Chen H., Wang Q., Zhao J., Ren X., Li D., Awasthi S.K., Shen F., Li R. (2017). Heterogeneity of Biochar Amendment to Improve the Carbon and Nitrogen Sequestration through Reduce the Greenhouse Gases Emissions during Sewage Sludge Composting. Bioresour. Technol..

[9] Gupta S., Sireesha S., Sreedhar I., Patel C.M., Anitha K.L. (2020). Latest Trends in Heavy Metal Removal from Wastewater by Biochar Based Sorbents. J. Water Process Eng..

[10] Zhao F., Tang L., Jiang H., Mao Y., Song W., Chen H. (2023). Prediction of Heavy Metals Adsorption by Hydrochars and Identification of Critical Factors Using Machine Learning Algorithms. Bioresour. Technol..

[11] Min L., Zhongsheng Z., Zhe L., Haitao W. (2020). Removal of Nitrogen and Phosphorus Pollutants from Water by FeCl3- Impregnated Biochar. Ecol. Eng..

[12] Premarathna K.S.D., Rajapaksha A.U., Sarkar B., Kwon E.E., Bhatnagar A., Ok Y.S., Vithanage M. (2019). Biochar-Based Engineered Composites for Sorptive Decontamination of Water: A Review. Chem. Eng. J..

[13] Chauhan S., Shafi T., Dubey B.K., Chowdhury S. (2023). Biochar-Mediated Removal of Pharmaceutical Compounds from Aqueous Matrices via Adsorption. Waste Dispos. Sustain. Energy.

[14] Manikandan S.K., Pallavi P., Shetty K., Bhattacharjee D., Giannakoudakis D.A., Katsoyiannis I.A., Nair V. (2023). Effective Usage of Biochar and Microorganisms for the Removal of Heavy Metal Ions and Pesticides. Molecules.

[15] Valizadeh S., Lee S.S., Choi Y.J., Baek K., Jeon B.-H., Andrew Lin K.-Y., Park Y.-K. (2022). Biochar Application Strategies for Polycyclic Aromatic Hydrocarbons Removal from Soils. Environ. Res..

[16] Saeed M., Ilyas N., Jayachandran K., Gaffar S., Arshad M., Sheeraz Ahmad M., Bibi F., Jeddi K., Hessini K. (2021). Biostimulation Potential of Biochar for Remediating the Crude Oil Contaminated Soil and Plant Growth. Saudi J. Biol. Sci..

[17] He P., Yu Q., Zhang H., Shao L., Lü F. (2017). Removal of Copper (II) by Biochar Mediated by Dissolved Organic Matter. Sci. Rep..

[18] Beesley L., Moreno-Jiménez E., Gomez-Eyles J.L., Harris E., Robinson B., Sizmur T. (2011). A Review of Biochars’ Potential Role in the Remediation, Revegetation and Restoration of Contaminated Soils. Environ. Pollut..

[19] Jeffery S., Verheijen F.G.A., van der Velde M., Bastos A.C. (2011). A Quantitative Review of the Effects of Biochar Application to Soils on Crop Productivity Using Meta-Analysis. Agric. Ecosyst. Environ..

[20] Kinney T.J., Masiello C.A., Dugan B., Hockaday W.C., Dean M.R., Zygourakis K., Barnes R.T. (2012). Hydrologic Properties of Biochars Produced at Different Temperatures. Biomass Bioenergy.

[21] Qin Y., Li G., Gao Y., Zhang L., Ok Y.S., An T. (2018). Persistent Free Radicals in Carbon-Based Materials on Transformation of Refractory Organic Contaminants (ROCs) in Water: A Critical Review. Water Res..

[22] Vejerano E.P., Rao G., Khachatryan L., Cormier S.A., Lomnicki S. (2018). Environmentally Persistent Free Radicals: Insights on a New Class of Pollutants. Environ. Sci. Technol..

[23] Volpe R., Bermudez Menendez J.M., Ramirez Reina T., Volpe M., Messineo A., Millan M., Titirici M.-M. (2019). Free Radicals Formation on Thermally Decomposed Biomass. Fuel.

[24] Huang Y., Guo X., Ding Z., Chen Y., Hu X. (2020). Environmentally Persistent Free Radicals in Biochar Derived from Laminaria Japonica Grown in Different Habitats. J. Anal. Appl. Pyrolysis.

[25] Ruan X., Sun Y., Du W., Tang Y., Liu Q., Zhang Z., Doherty W., Frost R.L., Qian G., Tsang D.C.W. (2019). Formation, Characteristics, and Applications of Environmentally Persistent Free Radicals in Biochars: A Review. Bioresour. Technol..

[26] Wang R.-Z., Huang D.-L., Liu Y.-G., Zhang C., Lai C., Wang X., Zeng G.-M., Gong X.-M., Duan A., Zhang Q. (2019). Recent Advances in Biochar-Based Catalysts: Properties, Applications and Mechanisms for Pollution Remediation. Chem. Eng. J..

[27] Odinga E.S., Waigi M.G., Gudda F.O., Wang J., Yang B., Hu X., Li S., Gao Y. (2020). Occurrence, Formation, Environmental Fate and Risks of Environmentally Persistent Free Radicals in Biochars. Environ. Int..

[28] Fang G., Liu C., Gao J., Dionysiou D.D., Zhou D. (2015). Manipulation of Persistent Free Radicals in Biochar to Activate Persulfate for Contaminant Degradation. Environ. Sci. Technol..

[29] Li F., Duan F., Ji W., Gui X. (2020). Biochar-activated persulfate for organic contaminants removal: Efficiency, mechanisms and influencing factors. Ecotoxicol. Environ. Saf..

[30] Liu F., Zhou H., Pan Z., Liu Y., Yao G., Guo Y., Lai B. (2020). Degradation of sulfamethoxazole by cobalt-nickel powder composite catalyst coupled with peroxymonosulfate: Performance, degradation pathways and mechanistic consideration. J. Hazard. Mater..

[31] Liang J., Xu X., Qamar Zaman W., Hu X., Zhao L., Qiu H., Cao X. (2019). Different Mechanisms between Biochar and Activated Carbon for the Persulfate Catalytic Degradation of Sulfamethoxazole: Roles of Radicals in Solution or Solid Phase. Chem. Eng. J..

[32] Fang G., Gao J., Liu C., Dionysiou D.D., Wang Y., Zhou D. (2014). Key Role of Persistent Free Radicals in Hydrogen Peroxide Activation by Biochar: Implications to Organic Contaminant Degradation. Environ. Sci. Technol..

[33] Fang G., Zhu C., Dionysiou D.D., Gao J., Zhou D. (2015). Mechanism of Hydroxyl Radical Generation from Biochar Suspensions: Implications to Diethyl Phthalate Degradation. Bioresour. Technol..

[34] Fang G., Liu C., Wang Y., Dionysiou D.D., Zhou D. (2017). Photogeneration of Reactive Oxygen Species from Biochar Suspension for Diethyl Phthalate Degradation. Appl. Catal. B Environ..

[35] Luo K., Yang Q., Pang Y., Wang D., Li X., Lei M., Huang Q. (2019). Unveiling the Mechanism of Biochar-Activated Hydrogen Peroxide on the Degradation of Ciprofloxacin. Chem. Eng. J..

[36] Zhang Y., Xu M., He R., Zhao J., Kang W., Lv J. (2022). Effect of Pyrolysis Temperature on the Activated Permonosulfate Degradation of Antibiotics in Nitrogen and Sulfur-Doping Biochar: Key Role of Environmentally Persistent Free Radicals. Chemosphere.

[37] Liao S., Pan B., Li H., Zhang D., Xing B. (2014). Detecting Free Radicals in Biochars and Determining Their Ability to Inhibit the Germination and Growth of Corn, Wheat and Rice Seedlings. Environ. Sci. Technol..

[38] Ahmad M., Rajapaksha A.U., Lim J.E., Zhang M., Bolan N., Mohan D., Vithanage M., Lee S.S., Ok Y.S. (2014). Biochar as a Sorbent for Contaminant Management in Soil and Water: A Review. Chemosphere.

[39] Qin J., Chen Q., Sun M., Sun P., Shen G. (2017). Pyrolysis Temperature-Induced Changes in the Catalytic Characteristics of Rice Husk-Derived Biochar during 1,3-Dichloropropene Degradation. Chem. Eng. J..

[40] Yang J., Pignatello J.J., Pan B., Xing B. (2017). Degradation of P-Nitrophenol by Lignin and Cellulose Chars: H_2_O_2_-Mediated Reaction and Direct Reaction with the Char. Environ. Sci. Technol..

[41] Zhang R., Zimmerman A.R., Zhang R., Li P., Zheng Y., Gao B. (2024). Persistent free radicals generated from a range of biochars and their physiological effects on wheat seedlings. Sci. Total Environ..

[42] Alfei S., Pandoli O.G. (2024). Bamboo-Based Biochar: A Still Too Little-Studied Black Gold and Its Current Applications. J. Xenobiot..

[43] Ginoble Pandoli O., Santos de Sá D., Nogueira Barbosa Junior M., Paciornik S., Palombini F.L., Nogueira F.M. (2023). Bamboo-Based Lignocellulose Biomass as Catalytic Support for Organic Synthesis and Water Treatments. Bamboo Science and Technology. Environmental Footprints and Eco-Design of Products and Processes.

[44] Liu X., Chen Z., Lu S., Shi X., Qu F., Cheng D., Wei W., Shon H.K., Ni B.J. (2024). Persistent free radicals on biochar for its catalytic capability: A review. Water Res..

[45] Luo K., Pang Y., Wang D., Li X., Wang L., Lei M., Huang Q., Yang Q. (2021). A critical review on the application of biochar in environmental pollution remediation: Role of persistent free radicals (PFRs). J. Environ. Sci..

[46] Yi J.F., Lin Z.Z., Li X., Zhou Y.Q., Guo Y. (2023). A short review on environmental distribution and toxicity of the environmentally persistent free radicals. Chemosphere.

[47] Jahirul M.I., Rasul M.G., Chowdhury A.A., Ashwath N. (2012). Biofuels Production through Biomass Pyrolysis—A Technological Review. Energies.

[48] Yaashikaa P.R., Senthil Kumar P., Varjani S.J., Saravanan A. (2019). A Review on Photochemical, Biochemical and Electrochemical Transformation of CO_2_ into Value-Added Products. J. CO2 Util..

[49] Saidur R., Abdelaziz E.A., Demirbas A., Hossain M.S., Mekhilef S. (2011). A Review on Biomass as a Fuel for Boilers. Renew. Sustain. Energy Rev..

[50] Yaashikaa P.R., Kumar P.S., Varjani S., Saravanan A. (2020). A Critical Review on the Biochar Production Techniques, Characterization, Stability and Applications for Circular Bioeconomy. Biotechnol. Rep..

[51] Lehmann J. (2009). Biochar for environmental management: An introduction. Biochar for Environmental Management. Sci. Technol..

[52] Mohanty S.K., Valenca R., Berger A.W., Yu I.K.M., Xiong X., Saunders T.M., Tsang D.C.W. (2018). Plenty of Room for Carbon on the Ground: Potential Applications of Biochar for Stormwater Treatment. Sci. Total Environ..

[53] Ayla Norris Smidth (2023). A New Boost for Biochar as a Natural Climate Solution. https://blog.nature.org/science-brief/a-new-boost-for-biochar-as-a-natural-climate-solution/#:~:text=Biochar%20is%20a%20type%20of%20charcoal%20made%20from,other%20biomass%20would%2C%20leading%20to%20more%20carbon%20sequestration.

[54] Yu D., Yu Y., Tang J., Li X., Ke C., Yao Z. (2022). Application Fields of Kitchen Waste Biochar and Its Prospects as Catalytic Material: A Review. Sci. Total Environ..

[55] Yu S., Zhang W., Dong X., Wang F., Yang W., Liu C., Chen D. (2024). A Review on Recent Advances of Biochar from Agricultural and Forestry Wastes: Preparation, Modification and Applications in Wastewater Treatment. J. Environ. Chem. Eng..

[56] Wijitkosum S. (2022). Biochar Derived from Agricultural Wastes and Wood Residues for Sustainable Agricultural and Environmental Applications. Int. Soil Water Conserv. Res..

[57] Yao Y., Gao B., Inyang M., Zimmerman A.R., Cao X., Pullammanappallil P., Yang L. (2011). Biochar Derived from Anaerobically Digested Sugar Beet Tailings: Characterization and Phosphate Removal Potential. Bioresour. Technol..

[58] Puettmann M., Sahoo K., Wilson K., Oneil E. (2020). Life Cycle Assessment of Biochar Produced from Forest Residues Using Portable Systems. J. Clean. Prod..

[59] Papageorgiou A., Azzi E.S., Enell A., Sundberg C. (2021). Biochar Produced from Wood Waste for Soil Remediation in Sweden: Carbon Sequestration and Other Environmental Impacts. Sci. Total Environ..

[60] Laird D. (2023). Biochar Amendments Make the Harvesting of Crop Residue for Bioenergy Production Sustainable. Nutr. Cycl. Agroecosystems.

[61] Li N., He M., Lu X., Yan B., Duan X., Chen G., Wang S., Hou L. (2022). Municipal Solid Waste Derived Biochars for Wastewater Treatment: Production, Properties and Applications. Resour. Conserv. Recycl..

[62] Shi W., Wang H., Yan J., Shan L., Quan G., Pan X., Cui L. (2022). Wheat Straw Derived Biochar with Hierarchically Porous Structure for Bisphenol A Removal: Preparation, Characterization, and Adsorption Properties. Sep. Purif. Technol..

[63] Foong S.Y., Chan Y.H., Chin B.L.F., Lock S.S.M., Yee C.Y., Yiin C.L., Peng W., Lam S.S. (2022). Production of Biochar from Rice Straw and Its Application for Wastewater Remediation—An Overview. Bioresour. Technol..

[64] Gunamantha M., Widana G.A.B. (2018). Characterization the Potential of Biochar from Cow and Pig Manure for Geoecology Applicationion. IOP Conf. Ser. Earth Environ. Sci..

[65] Rathnayake D., Schmidt H.-P., Leifeld J., Mayer J., Epper C.A., Bucheli T.D., Hagemann N. (2023). Biochar from Animal Manure: A Critical Assessment on Technical Feasibility, Economic Viability, and Ecological Impact. GCB Bioenergy.

[66] Drané M., Zbair M., Hajjar-Garreau S., Josien L., Michelin L., Bennici S., Limousy L. (2024). Unveiling the Potential of Corn Cob Biochar: Analysis of Microstructure and Composition with Emphasis on Interaction with NO_2_. Materials.

[67] Luo L., Wang J., Lv J., Liu Z., Sun T., Yang Y., Zhu Y.-G. (2023). Carbon Sequestration Strategies in Soil Using Biochar: Advances, Challenges, and Opportunities. Environ. Sci. Technol..

[68] Brassard P., Godbout S., Lévesque V., Palacios J.H., Raghavan V., Ahmed A., Hogue R., Jeanne T., Verma M., Jeguirim M., Limousy L. (2019). 4—Biochar for Soil Amendment. Char and Carbon Materials Derived from Biomass.

[69] Godlewska P., Schmidt H.P., Ok Y.S., Oleszczuk P. (2017). Biochar for Composting Improvement and Contaminants Reduction. A Review. Bioresour. Technol..

[70] Xiang W., Zhang X., Chen J., Zou W., He F., Hu X., Tsang D.C.W., Ok Y.S., Gao B. (2020). Biochar Technology in Wastewater Treatment: A Critical Review. Chemosphere.

[71] Rawat S., Wang C.-T., Lay C.-H., Hotha S., Bhaskar T. (2023). Sustainable Biochar for Advanced Electrochemical/Energy Storage Applications. J. Energy Storage.

[72] Qiu B., Shao Q., Shi J., Yang C., Chu H. (2022). Application of Biochar for the Adsorption of Organic Pollutants from Wastewater: Modification Strategies, Mechanisms and Challenges. Sep. Purif. Technol..

[73] Kalu S., Kulmala L., Zrim J., Peltokangas K., Tammeorg P., Rasa K., Kitzler B., Pihlatie M., Karhu K. (2022). Potential of Biochar to Reduce Greenhouse Gas Emissions and Increase Nitrogen Use Efficiency in Boreal Arable Soils in the Long-Term. Front. Environ. Sci..

[74] Jiang T., Wang B., Gao B., Cheng N., Feng Q., Chen M., Wang S. (2023). Degradation of Organic Pollutants from Water by Biochar-Assisted Advanced Oxidation Processes: Mechanisms and Applications. J. Hazard. Mater..

[75] Lyu H., Zhang Q., Shen B. (2020). Application of Biochar and Its Composites in Catalysis. Chemosphere.

[76] Gayathri R., Gopinath K.P., Kumar P.S. (2021). Adsorptive Separation of Toxic Metals from Aquatic Environment Using Agro Waste Biochar: Application in Electroplating Industrial Wastewater. Chemosphere.

[77] Hemavathy R.V., Kumar P.S., Kanmani K., Jahnavi N. (2020). Adsorptive Separation of Cu(II) Ions from Aqueous Medium Using Thermally/Chemically Treated Cassia Fistula Based Biochar. J. Clean. Prod..

[78] El-Naggar A., El-Naggar A.H., Shaheen S.M., Sarkar B., Chang S.X., Tsang D.C.W., Rinklebe J., Ok Y.S. (2019). Biochar Composition-Dependent Impacts on Soil Nutrient Release, Carbon Mineralization, and Potential Environmental Risk: A Review. J. Environ. Manag..

[79] El-Naggar A., Lee S.S., Rinklebe J., Farooq M., Song H., Sarmah A.K., Zimmerman A.R., Ahmad M., Shaheen S.M., Ok Y.S. (2019). Biochar Application to Low Fertility Soils: A Review of Current Status, and Future Prospects. Geoderma.

[80] Hu X., Xu J., Wu M., Xing J., Bi W., Wang K., Ma J., Liu X. (2017). Effects of Biomass Pre-Pyrolysis and Pyrolysis Temperature on Magnetic Biochar Properties. J. Anal. Appl. Pyrolysis.

[81] Senthil Kumar P., Abhinaya R.V., Gayathri Lashmi K., Arthi V., Pavithra R., Sathyaselvabala V., Dinesh Kirupha S., Sivanesan S. (2011). Adsorption of Methylene Blue Dye from Aqueous Solution by Agricultural Waste: Equilibrium, Thermodynamics, Kinetics, Mechanism and Process Design. Colloid J..

[82] Kumar P.S., Senthamarai C., Deepthi A.S.L.S., Bharani R. (2013). Adsorption Isotherms, Kinetics and Mechanism of Pb(II) Ions Removal from Aqueous Solution Using Chemically Modified Agricultural Waste. Can. J. Chem. Eng..

[83] Varjani S., Kumar G., Rene E.R. (2019). Developments in Biochar Application for Pesticide Remediation: Current Knowledge and Future Research Directions. J. Environ. Manag..

[84] Bridgwater A.V. (2012). Review of Fast Pyrolysis of Biomass and Product Upgrading. Biomass Bioenergy.

[85] Ng W.C., You S., Ling R., Gin K.Y.-H., Dai Y., Wang C.-H. (2017). Co-Gasification of Woody Biomass and Chicken Manure: Syngas Production, Biochar Reutilization, and Cost-Benefit Analysis. Energy.

[86] Cantrell K.B., Hunt P.G., Uchimiya M., Novak J.M., Ro K.S. (2012). Impact of Pyrolysis Temperature and Manure Source on Physicochemical Characteristics of Biochar. Bioresour. Technol..

[87] Funke A., Ziegler F. (2010). Hydrothermal Carbonization of Biomass: A Summary and Discussion of Chemical Mechanisms for Process Engineering. Biofuels Bioprod. Biorefining.

[88] Klinghoffer N.B., Castaldi M.J., Nzihou A. (2015). Influence of Char Composition and Inorganics on Catalytic Activity of Char from Biomass Gasification. Fuel.

[89] Bergman P.C., Boersma A.R., Zwart R.W.R., Kiel J.H. (2005). Torrefaction for Biomass Co-Firing in Existing Coal-Fired Power Stations.

[90] Nunoura T., Wade S.R., Bourke J.P., Antal M.J. (2006). Studies of the Flash Carbonization Process. 1. Propagation of the Flaming Pyrolysis Reaction and Performance of a Catalytic Afterburner. Ind. Eng. Chem. Res..

[91] Basu P., Basu P. (2018). Chapter 5—Pyrolysis. Biomass Gasification, Pyrolysis and Torrefaction.

[92] Devi M., Rawat S., Sharma S. (2021). A Comprehensive Review of the Pyrolysis Process: From Carbon Nanomaterial Synthesis to Waste Treatment. Oxf. Open Mater. Sci..

[93] Cha J.S., Park S.H., Jung S.-C., Ryu C., Jeon J.-K., Shin M.-C., Park Y.-K. (2016). Production and Utilization of Biochar: A Review. J. Ind. Eng. Chem..

[94] Yaashikaa P.R., Senthil Kumar P., Varjani S.J., Saravanan A. (2019). Advances in Production and Application of Biochar from Lignocellulosic Feedstocks for Remediation of Environmental Pollutants. Bioresour. Technol..

[95] Neogi S., Sharma V., Khan N., Chaurasia D., Ahmad A., Chauhan S., Singh A., You S., Pandey A., Bhargava P.C. (2022). Sustainable biochar: A facile strategy for soil and environmental restoration, energy generation, mitigation of global climate change and circular bioeconomy. Chemosphere.

[96] Swagathnath G., Rangabhashiyam S., Parthsarathi K., Murugan S., Balasubramanian P., Drück H., Pillai R.G., Tharian M.G., Majeed A.Z. (2019). Modeling Biochar Yield and Syngas Production During the Pyrolysis of Agro-Residues. Proceedings of the Green Buildings and Sustainable Engineering.

[97] Tan H., Lee C.T., Ong P.Y., Wong K.Y., Bong CP C., Li C., Gao Y. (2021). A Review on the Comparison Between Slow Pyrolysis and Fast Pyrolysis on the Quality of Lignocellulosic and Lignin-Based Biochar. IOP Conf. Ser. Mater. Sci. Eng..

[98] Ibitoye S.E., Mahamood R.M., Jen T.-C., Loha C., Akinlabi E.T. (2023). An Overview of Biomass Solid Fuels: Biomass Sources, Processing Methods, and Morphological and Microstructural Properties. J. Bioresour. Bioprod..

[99] Kumar A., Bhattacharya T. Biochar and its application. Proceedings of the Conference: Biogeochemical Cycles and Climate Change.

[100] Tomczyk A., Sokołowska Z., Boguta P. (2020). Biochar Physicochemical Properties: Pyrolysis Temperature and Feedstock Kind Effects. Rev. Environ. Sci. Bio/Technol..

[101] Ding W., Dong X., Ime I.M., Gao B., Ma L.Q. (2014). Pyrolytic Temperatures Impact Lead Sorption Mechanisms by Bagasse Biochars. Chemosphere.

[102] Banik C., Lawrinenko M., Bakshi S., Laird D.A. (2018). Impact of Pyrolysis Temperature and Feedstock on Surface Charge and Functional Group Chemistry of Biochars. J. Environ. Qual..

[103] Zhao S.-X., Ta N., Wang X.-D. (2017). Effect of Temperature on the Structural and Physicochemical Properties of Biochar with Apple Tree Branches as Feedstock Material. Energies.

[104] Suliman W., Harsh J.B., Abu-Lail N.I., Fortuna A.-M., Dallmeyer I., Garcia-Pérez M. (2017). The Role of Biochar Porosity and Surface Functionality in Augmenting Hydrologic Properties of a Sandy Soil. Sci. Total Environ..

[105] Gontijo L.O., Junior M.N.B., de Sá D.S., Letichevsky S., Pedrozo-Peñafiel M.J., Aucélio R.Q., Bott I.S., Alves H.D.L., Fragneaud B., Maciel I.O. (2023). 3D conductive monolithic carbons from pyrolyzed bamboo for microfluidic self-heating system. Carbon.

[106] Sudarsan J.S., Prasanna K., Shiam Babu R., Sai Krishna V.M.V., Ravindran B., Gupta S.K., Bhat S.A., Chauhan P.S., Tyagi N. (2023). Chapter 11—Biochar: A Sustainable Solution for Organic Waste Management a Way Forward towards Circular Economy. Recent Trends in Solid Waste Management.

[107] Zhou B., Liu Q., Shi L., Liu Z. (2019). Electron Spin Resonance Studies of Coals and Coal Conversion Processes: A Review. Fuel Process. Technol..

[108] Zhang S., Cui J., Wu H., Zheng Q., Song D., Wang X., Zhang S. (2021). Organic Carbon, Total Nitrogen, and Microbial Community Distributions within Aggregates of Calcareous Soil Treated with Biochar. Agric. Ecosyst. Environ..

[109] Ma L., Song D., Liu M., Li Y., Li Y. (2022). Effects of Earthworm Activities on Soil Nutrients and Microbial Diversity under Different Tillage Measures. Soil Tillage Res..

[110] Hu W., Gao W., Tang Y., Zhang Q., Tu C., Cheng J. (2022). Remediation via Biochar and Potential Health Risk of Heavy Metal Contaminated Soils. Environ. Earth Sci..

[111] Kant Bhatia S., Palai A.K., Kumar A., Kant Bhatia R., Kumar Patel A., Kumar Thakur V., Yang Y.-H. (2021). Trends in Renewable Energy Production Employing Biomass-Based Biochar. Bioresour. Technol..

[112] Sakhiya A.K., Anand A., Kaushal P. (2020). Production, Activation, and Applications of Biochar in Recent Times. Biochar.

[113] Nguyen H.N., Pignatello J.J. (2013). Laboratory Tests of Biochars as Absorbents for Use in Recovery or Containment of Marine Crude Oil Spills. Environ. Eng. Sci..

[114] Jothirani R., Kumar P.S., Saravanan A., Narayan A.S., Dutta A. (2016). Ultrasonic Modified Corn Pith for the Sequestration of Dye from Aqueous Solution. J. Ind. Eng. Chem..

[115] Suganya S., Senthil Kumar P., Saravanan A., Sundar Rajan P., Ravikumar C. (2017). Computation of Adsorption Parameters for the Removal of Dye from Wastewater by Microwave Assisted Sawdust: Theoretical and Experimental Analysis. Environ. Toxicol. Pharmacol..

[116] Saravanan A., Kumar P.S., Renita A.A. (2018). Hybrid Synthesis of Novel Material through Acid Modification Followed Ultrasonication to Improve Adsorption Capacity for Zinc Removal. J. Clean. Prod..

[117] Luo Z., Yao B., Yang X., Wang L., Xu Z., Yan X., Tian L., Zhou H., Zhou Y. (2022). Novel Insights into the Adsorption of Organic Contaminants by Biochar: A Review. Chemosphere.

[118] Lou L., Wu B., Wang L., Luo L., Xu X., Hou J., Xun B., Hu B., Chen Y. (2011). Sorption and Ecotoxicity of Pentachlorophenol Polluted Sediment Amended with Rice-Straw Derived Biochar. Bioresour. Technol..

[119] Tan X., Liu Y., Zeng G., Wang X., Hu X., Gu Y., Yang Z. (2015). Application of Biochar for the Removal of Pollutants from Aqueous Solutions. Chemosphere.

[120] Yu X.-Y., Ying G.-G., Kookana R.S. (2009). Reduced Plant Uptake of Pesticides with Biochar Additions to Soil. Chemosphere.

[121] Liang C., Niu H.-Y., Guo H., Niu C.-G., Yang Y.-Y., Liu H.-Y., Tang W.-W., Feng H.-P. (2021). Efficient Photocatalytic Nitrogen Fixation to Ammonia over Bismuth Monoxide Quantum Dots-Modified Defective Ultrathin Graphitic Carbon Nitride. Chem. Eng. J..

[122] Kumbhar D., Palliyarayil A., Reghu D., Shrungar D., Umapathy S., Sil S. (2021). Rapid Discrimination of Porous Bio-Carbon Derived from Nitrogen Rich Biomass Using Raman Spectroscopy and Artificial Intelligence Methods. Carbon.

[123] Zhang K., Sun P., Faye M.C.A.S., Zhang Y. (2018). Characterization of Biochar Derived from Rice Husks and Its Potential in Chlorobenzene Degradation. Carbon.

[124] Chen D., Xu J., Ling P., Fang Z., Ren Q., Xu K., Jiang L., Wang Y., Su S., Hu S. (2024). Formation and Evolution Mechanism of Persistent Free Radicals in Biochar during Biomass Pyrolysis: Insights from Biochar’s Element Composition and Chemical Structure. Fuel.

[125] Wu C., Fu L., Li H., Liu X., Wan C. (2022). Using Biochar to Strengthen the Removal of Antibiotic Resistance Genes: Performance and Mechanism. Sci. Total Environ..

[126] Huang C., Qin F., Zhang C., Huang D., Tang L., Yan M., Wang W., Song B., Qin D., Zhou Y. (2023). Effects of Heterogeneous Metals on the Generation of Persistent Free Radicals as Critical Redox Sites in Iron-Containing Biochar for Persulfate Activation. ACS EST Water.

[127] Yu J., Zhu Z., Zhang H., Shen X., Qiu Y., Yin D., Wang S. (2020). Persistent Free Radicals on N-Doped Hydrochar for Degradation of Endocrine Disrupting Compounds. Chem. Eng. J..

[128] Ma L., Syed-Hassan S.S.A., Tong Y., Xiong Z., Chen Y., Xu J., Jiang L., Su S., Hu S., Wang Y. (2023). Interactions of Cellulose- and Lignin-Derived Radicals during Pyrolysis: An in-Situ Electron Paramagnetic Resonance (EPR) Study. Fuel Process. Technol..

[129] Retcofsky H.L., Hough M.R., Clarkson R.B. (1979). Nature of the Free Radicals in Coals, Pyrolyzed Coals, and Liquefaction Products. Am. Chem. Soc. Div. Fuel Chem. Prepr..

[130] Liu J., Jiang X., Shen J., Zhang H. (2014). Chemical Properties of Superfine Pulverized Coal Particles. Part 1. Electron Paramagnetic Resonance Analysis of Free Radical Characteristics. Adv. Powder Technol..

[131] Zhang Y., Sun X., Bian W., Peng J., Wan H., Zhao J. (2020). The Key Role of Persistent Free Radicals on the Surface of Hydrochar and Pyrocarbon in the Removal of Heavy Metal-Organic Combined Pollutants. Bioresour. Technol..

[132] Qin J., Cheng Y., Sun M., Yan L., Shen G. (2016). Catalytic Degradation of the Soil Fumigant 1,3-Dichloropropene in Aqueous Biochar Slurry. Sci. Total Environ..

[133] Huang D., Luo H., Zhang C., Zeng G., Lai C., Cheng M., Wang R., Deng R., Xue W., Gong X. (2019). Nonnegligible Role of Biomass Types and Its Compositions on the Formation of Persistent Free Radicals in Biochar: Insight into the Influences on Fenton-like Process. Chem. Eng. J..

[134] Wang Y., Gu X., Huang Y., Ding Z., Chen Y., Hu X. (2022). Insight into Biomass Feedstock on Formation of Biochar-Bound Environmentally Persistent Free Radicals under Different Pyrolysis Temperatures. RSC Adv..

[135] Tao W., Duan W., Liu C., Zhu D., Si X., Zhu R., Oleszczuk P., Pan B. (2020). Formation of Persistent Free Radicals in Biochar Derived from Rice Straw Based on a Detailed Analysis of Pyrolysis Kinetics. Sci. Total Environ..

[136] Xiang C., Liu Q., Shi L., Liu Z. (2020). A Study on the New Type of Radicals in Corncob Derived Biochars. Fuel.

[137] Chintala R., Subramanian S., Fortuna A.-M., Schumacher T.E., Komang Ralebitso-Senior T., Orr C.H. (2016). Examining Biochar Impacts on Soil Abiotic and Biotic Processes and Exploring the Potential for Pyrosequencing Analysis. Chapter 6. Biochar Application Essential Soil Microbial Ecology.

[138] Takeshita A., Uemura Y., Onoe K. (2020). Quantification of Persistent Free Radicals (PFRs) Formed in Thermally Decomposed Cellulose and Its Correlation with Residual Carbon Amount. J. Anal. Appl. Pyrolysis.

[139] Pan B., Li H., Lang D., Xing B. (2019). Environmentally Persistent Free Radicals: Occurrence, Formation Mechanisms and Implications. Environ. Pollut..

[140] Lomnicki S.M., Truong H., Vejerano E.P., Dellinger B. (2008). Copper Oxide-Based Model of Persistent Free Radical Formation on Combustion-Derived Particulate Matter. Environ. Sci. Technol..

[141] Gasim M.F., Choong Z.-Y., Koo P.-L., Low S.-C., Abdurahman M.-H., Ho Y.-C., Mohamad M., Suryawan I.W., Lim J.-W., Oh W.-D. (2022). Application of Biochar as Functional Material for Remediation of Organic Pollutants in Water: An Overview. Catalysts.

[142] Zhong D., Jiang Y., Zhao Z., Wang L., Chen J., Ren S., Liu Z., Zhang Y., Tsang D.C.W., Crittenden J.C. (2019). pH Dependence of Arsenic Oxidation by Rice-Husk-Derived Biochar: Roles of Redox-Active Moieties. Environ. Sci. Technol..

[143] Zhang K., Sun P., Zhang Y. (2019). Decontamination of Cr(VI) Facilitated Formation of Persistent Free Radicals on Rice Husk Derived Biochar. Front. Environ. Sci. Eng..

[144] Zhu S., Huang X., Yang X., Peng P., Li Z., Jin C. (2020). Enhanced Transformation of Cr(VI) by Heterocyclic-N within Nitrogen-Doped Biochar: Impact of Surface Modulatory Persistent Free Radicals (PFRs). Environ. Sci. Technol..

[145] Wang X., Xu J., Liu J., Liu J., Xia F., Wang C., Dahlgren R.A., Liu W. (2020). Mechanism of Cr(VI) Removal by Magnetic Greigite/Biochar Composites. Sci. Total Environ..

[146] Tang Z., Kong Y., Zhao S., Jia H., Vione D., Kang Y., Gao P. (2021). Enhancement of Cr(VI) Decontamination by Irradiated Sludge Biochar in Neutral Conditions: Evidence of a Possible Role of Persistent Free Radicals. Sep. Purif. Technol..

[147] Zhu Y., Wei J., Li J. (2023). Decontamination of Cr(VI) from Water Using Sewage Sludge-Derived Biochar: Role of Environmentally Persistent Free Radicals. Chin. J. Chem. Eng..

[148] Baltrėnaitė-Gedienė E., Lomnicki S., Guo C. (2022). Impact of Biochar, Fertilizers and Cultivation Type on Environmentally Persistent Free Radicals in Agricultural Soil. Environ. Technol. Innov..

[149] Tai Y., Sun J., Tian H., Liu F., Han B., Fu W., Liu Z., Yang X., Liu Q. (2023). Efficient Degradation of Organic Pollutants by S-NaTaO_3_/Biochar under Visible Light and the Photocatalytic Performance of a Permonosulfate-Based Dual-Effect Catalytic System. J. Environ. Sci..

[150] Kelley M.A., Hebert V.Y., Thibeaux T.M., Orchard M.A., Hasan F., Cormier S.A., Thevenot P.T., Lomnicki S.M., Varner K.J., Dellinger B. (2013). Model Combustion-Generated Particulate Matter Containing Persistent Free Radicals Redox Cycle to Produce Reactive Oxygen Species. Chem. Res. Toxicol..

[151] Squadrito G.L., Cueto R., Dellinger B., Pryor W.A. (2001). Quinoid Redox Cycling as a Mechanism for Sustained Free Radical Generation by Inhaled Airborne Particulate Matter. Free Radic. Biol. Med..

[152] Alburquerque J.A., Salazar P., Barrón V., Torrent J., del Campillo M.d.C., Gallardo A., Villar R. (2013). Enhanced Wheat Yield by Biochar Addition under Different Mineral Fertilization Levels. Agron. Sustain. Dev..

[153] Jiang S.-F., Ling L.-L., Chen W.-J., Liu W.-J., Li D.-C., Jiang H. (2019). High Efficient Removal of Bisphenol A in a Peroxymonosulfate/Iron Functionalized Biochar System: Mechanistic Elucidation and Quantification of the Contributors. Chem. Eng. J..

[154] Luo H., Lin Q., Zhang X., Huang Z., Liu S., Jiang J., Xiao R., Liao X. (2019). New Insights into the Formation and Transformation of Active Species in nZVI/BC Activated Persulfate in Alkaline Solutions. Chem. Eng. J..

[155] Pi Z., Li X., Wang D., Xu Q., Tao Z., Huang X., Yao F., Wu Y., He L., Yang Q. (2019). Persulfate Activation by Oxidation Biochar Supported Magnetite Particles for Tetracycline Removal: Performance and Degradation Pathway. J. Clean. Prod..

[156] Zhang X., Huang R., Show P.L., Mahlknecht J., Wang C. (2024). Degradation of Tetracycline by Nitrogen-Doped Biochar as a Peroxydisulfate Activator: Nitrogen Doping Pattern and Non-Radical Mechanism. Sustain. Horiz..

[157] He J., Xiao Y., Tang J., Chen H., Sun H. (2019). Persulfate Activation with Sawdust Biochar in Aqueous Solution by Enhanced Electron Donor-Transfer Effect. Sci. Total Environ..

[158] Danping W.U., Fangfang L.I., Jing Z., Peng W., Min W.U. (2019). Photocatalysis degradation of rhodamine B by dissolved organic matter of biochars. Chin. J. Environ. Eng..

[159] Wang H., Guo W., Yin R., Du J., Wu Q., Luo H., Liu B., Sseguya F., Ren N. (2019). Biochar-Induced Fe(III) Reduction for Persulfate Activation in Sulfamethoxazole Degradation: Insight into the Electron Transfer, Radical Oxidation and Degradation Pathways. Chem. Eng. J..

[160] Zhu K., Wang X., Geng M., Chen D., Lin H., Zhang H. (2019). Catalytic Oxidation of Clofibric Acid by Peroxydisulfate Activated with Wood-Based Biochar: Effect of Biochar Pyrolysis Temperature, Performance and Mechanism. Chem. Eng. J..

[161] Li L., Lai C., Huang F., Cheng M., Zeng G., Huang D., Li B., Liu S., Zhang M., Qin L. (2019). Degradation of Naphthalene with Magnetic Bio-Char Activate Hydrogen Peroxide: Synergism of Bio-Char and Fe–Mn Binary Oxides. Water Res..

[162] Luo K., Pang Y., Yang Q., Wang D., Li X., Wang L., Lei M., Liu J. (2019). Enhanced Ciprofloxacin Removal by Sludge-Derived Biochar: Effect of Humic Acid. Chemosphere.

[163] Deng R., Luo H., Huang D., Zhang C. (2020). Biochar-Mediated Fenton-like Reaction for the Degradation of Sulfamethazine: Role of Environmentally Persistent Free Radicals. Chemosphere.

[164] Liu G., Zhang Y., Yu H., Jin R., Zhou J. (2020). Acceleration of Goethite-Catalyzed Fenton-like Oxidation of Ofloxacin by Biochar. J. Hazard. Mater..

[165] Liu B., Guo W., Wang H., Si Q., Zhao Q., Luo H., Ren N. (2020). Activation of Peroxymonosulfate by Cobalt-Impregnated Biochar for Atrazine Degradation: The Pivotal Roles of Persistent Free Radicals and Ecotoxicity Assessment. J. Hazard. Mater..

[166] Grilla E., Vakros J., Konstantinou I., Manariotis I.D., Mantzavinos D. (2020). Activation of Persulfate by Biochar from Spent Malt Rootlets for the Degradation of Trimethoprim in the Presence of Inorganic Ions. J. Chem. Technol. Biotechnol..

[167] Feng Z., Zhou B., Yuan R., Li H., He P., Wang F., Chen Z., Chen H. (2022). Biochar Derived from Different Crop Straws as Persulfate Activator for the Degradation of Sulfadiazine: Influence of Biomass Types and Systemic Cause Analysis. Chem. Eng. J..

[168] Kim D.-G., Ko S.-O. (2020). Effects of Thermal Modification of a Biochar on Persulfate Activation and Mechanisms of Catalytic Degradation of a Pharmaceutical. Chem. Eng. J..

[169] He W., Zhu Y., Zeng G., Zhang Y., Wang Y., Zhang M., Long H., Tang W. (2020). Efficient Removal of Perfluorooctanoic Acid by Persulfate Advanced Oxidative Degradation: Inherent Roles of Iron-Porphyrin and Persistent Free Radicals. Chem. Eng. J..

[170] Cao W., Zeng C., Guo X., Liu Q., Zhang X., Mameda N. (2020). Enhanced Electrochemical Degradation of 2,4-Dichlorophenol with the Assist of Hydrochar. Chemosphere.

[171] Xu H., Zhang Y., Li J., Hao Q., Li X., Liu F. (2020). Heterogeneous Activation of Peroxymonosulfate by a Biochar-Supported Co_3_O_4_ Composite for Efficient Degradation of Chloramphenicols. Environ. Pollut..

[172] Li X., Jia Y., Zhou M., Su X., Sun J. (2020). High-Efficiency Degradation of Organic Pollutants with Fe, N Co-Doped Biochar Catalysts via Persulfate Activation. J. Hazard. Mater..

[173] Xie Y., Hu W., Wang X., Tong W., Li P., Zhou H., Wang Y., Zhang Y. (2020). Molten Salt Induced Nitrogen-Doped Biochar Nanosheets as Highly Efficient Peroxymonosulfate Catalyst for Organic Pollutant Degradation. Environ. Pollut..

[174] Yan J., Yang L., Qian L., Han L., Chen M. (2020). Nano-Magnetite Supported by Biochar Pyrolyzed at Different Temperatures as Hydrogen Peroxide Activator: Synthesis Mechanism and the Effects on Ethylbenzene Removal. Environ. Pollut..

[175] Mian M.M., Liu G., Zhou H. (2020). Preparation of N-Doped Biochar from Sewage Sludge and Melamine for Peroxymonosulfate Activation: N-Functionality and Catalytic Mechanisms. Sci. Total Environ..

[176] da Silva Veiga P.A., Schultz J., da Silva Matos T.T., Fornari M.R., Costa T.G., Meurer L., Mangrich A.S. (2020). Production of High-Performance Biochar Using a Simple and Low-Cost Method: Optimization of Pyrolysis Parameters and Evaluation for Water Treatment. J. Anal. Appl. Pyrolysis.

[177] Sun P., Zhang K.-K., Zhang Y., Zhang Y. (2020). Sunflower-Straw-Derived Biochar-Enhanced Fe(III)/S_2_O_8_^2−^ System for Degradation of Benzoic Acid. Environ. Sci..

[178] Zeng L., Chen Q., Tan Y., Lan P., Zhou D., Wu M., Liang N., Pan B., Xing B. (2021). Dual Roles of Biochar Redox Property in Mediating 2,4-Dichlorophenol Degradation in the Presence of Fe^3+^ and Persulfate. Chemosphere.

[179] Yang F., Zhu Q., Gao Y., Jian H., Wang C., Sun H. (2021). Effects of Biochar-Dissolved Organic Matter on the Photodegradation of Sulfamethoxazole and Chloramphenicol in Biochar Solutions as Revealed by Oxygen Reduction Performances and Free Radicals. Sci. Total Environ..

[180] Min L., Zhang P., Fan M., Xu X., Wang C., Tang J., Sun H. (2021). Efficient Degradation of P-Nitrophenol by Fe@pomelo Peel-Derived Biochar Composites and Its Mechanism of Simultaneous Reduction and Oxidation Process. Chemosphere.

[181] Mer K., Sajjadi B., Egiebor N.O., Chen W.Y., Mattern D.L., Tao W. (2021). Enhanced Degradation of Organic Contaminants Using Catalytic Activity of Carbonaceous Structures: A Strategy for the Reuse of Exhausted Sorbents. J. Environ. Sci..

[182] Cao Y., Jiang S., Kang X., Zhang H., Zhang Q., Wang L. (2021). Enhancing Degradation of Atrazine by Fe-Phenol Modified Biochar/Ferrate(VI) under Alkaline Conditions: Analysis of the Mechanism and Intermediate Products. Chemosphere.

[183] Zhang R., Zheng X., Zhang D., Niu X., Ma J., Lin Z., Fu M., Zhou S. (2021). Insight into the Roles of Endogenous Minerals in the Activation of Persulfate by Graphitized Biochar for Tetracycline Removal. Sci. Total Environ..

[184] Yi Y., Luo J., Fang Z. (2021). Magnetic Biochar Derived from Eichhornia Crassipes for Highly Efficient Fenton-like Degradation of Antibiotics: Mechanism and Contributions. J. Environ. Chem. Eng..

[185] Zhang Y., Xu M., Liang S., Feng Z., Zhao J. (2021). Mechanism of Persulfate Activation by Biochar for the Catalytic Degradation of Antibiotics: Synergistic Effects of Environmentally Persistent Free Radicals and the Defective Structure of Biochar. Sci. Total Environ..

[186] Wang X., Zhang P., Wang C., Jia H., Shang X., Tang J., Sun H. (2022). Metal-Rich Hyperaccumulator-Derived Biochar as an Efficient Persulfate Activator: Role of Intrinsic Metals (Fe, Mn and Zn) in Regulating Characteristics, Performance and Reaction Mechanisms. J. Hazard. Mater..

[187] Luo K., Yang C., Li X., Pang Y., Yang Q. (2022). Mn-Doped Biochar Derived from Sewage Sludge for Ciprofloxacin Degradation. J. Environ. Eng..

[188] Li H., Liu Y., Jiang F., Bai X., Li H., Lang D., Wang L., Pan B. (2022). Persulfate Adsorption and Activation by Carbon Structure Defects Provided New Insights into Ofloxacin Degradation by Biochar. Sci. Total Environ..

[189] Dai Z., Zhao L., Peng S., Yue Z., Zhan X., Wang J. (2022). Removal of Oxytetracycline Promoted by Manganese-Doped Biochar Based on Density Functional Theory Calculations: Comprehensive Evaluation of the Effect of Transition Metal Doping. Sci. Total Environ..

[190] Rangarajan G., Farnood R. (2022). Role of Persistent Free Radicals and Lewis Acid Sites in Visible-Light-Driven Wet Peroxide Activation by Solid Acid Biochar Catalysts—A Mechanistic Study. J. Hazard. Mater..

[191] An X., Chen Y., Ao M., Jin Y., Zhan L., Yu B., Wu Z., Jiang P. (2022). Sequential Photocatalytic Degradation of Organophosphorus Pesticides and Recovery of Orthophosphate by Biochar/α-Fe_2_O_3_/MgO Composite: A New Enhanced Strategy for Reducing the Impacts of Organophosphorus from Wastewater. Chem. Eng. J..

[192] Jiang X., Xiao Y., Xiao J., Zhang W., Qiu R. The Effect of Persistent Free Radicals in Sludge Derived Biochar on the Removal of P-Chlorophenol. https://ssrn.com/abstract=3992617.

[193] Pan Y., Peng Z., Liu Z., Shao B., Liang Q., He Q., Wu T., Zhang X., Zhao C., Liu Y. (2022). Activation of Peroxydisulfate by Bimetal Modified Peanut Hull-Derived Porous Biochar for the Degradation of Tetracycline in Aqueous Solution. J. Environ. Chem. Eng..

[194] Yin Q., Yan H., Liang Y., Jiang Z., Wang H., Nian Y. (2023). Activation of Persulfate by Blue Algae Biochar Supported FeO_X_ Particles for Tetracycline Degradation: Performance and Mechanism. Sep. Purif. Technol..

[195] Badiger S.M., Nidheesh P.V. (2023). Applications of Biochar in Sulfate Radical-Based Advanced Oxidation Processes for the Removal of Pharmaceuticals and Personal Care Products. Water Sci. Technol..

[196] Yu Y., Zhong Z., Guo H., Yu Y., Zheng T., Li H., Chang Z. (2023). Biochar–Goethite Composites Inhibited/Enhanced Degradation of Triphenyl Phosphate by Activating Persulfate: Insights on the Mechanism. Sci. Total Environ..

[197] Pei S., Zhao Y., Li W., Qu C., Ren Y., Yang Y., Liu J., Wu C. (2023). Critical Impact of Pyrolysis Temperatures on Biochars for Peroxymonosulfate Activation: Structural Characteristics, Degradation Performance and Mechanism. Chem. Eng. J..

[198] Zhang Y., He R., Zhao J., Zhang X., Bildyukevich A.V. (2023). Effect of Aged Biochar after Microbial Fermentation on Antibiotics Removal: Key Roles of Microplastics and Environmentally Persistent Free Radicals. Bioresour. Technol..

[199] Yang Z., An Q., Deng S., Xu B., Li Z., Deng S., Zhao B., Ye Z. (2023). Efficient Activation of Peroxydisulfate by Modified Red Mud Biochar Derived from Waste Corn Straw for Levofloxacin Degradation: Efficiencies and Mechanisms. J. Environ. Chem. Eng..

[200] Zhang Y., He R., Zhao J. (2023). Removal Mechanism of Tetracycline-Cr(VI) Combined Pollutants by Different S-Doped Sludge Biochars: Role of Environmentally Persistent Free Radicals. Chemosphere.

[201] Luo K., Lin N., Li X., Pang Y., Wang L., Lei M., Yang Q. (2023). Efficient Hexavalent Chromium Removal by Nano-Cerium Oxide Functionalized Biochar: Insight into the Role of Reduction. J. Environ. Chem. Eng..

[202] Zhong D., Zhao Z., Jiang Y., Yang X., Wang L., Chen J., Guan C.-Y., Zhang Y., Tsang D.C.W., Crittenden J.C. (2020). Contrasting Abiotic As(III) Immobilization by Undissolved and Dissolved Fractions of Biochar in Ca^2+^-Rich Groundwater under Anoxic Conditions. Water Res..

[203] Huang Y., Gao M., Deng Y., Khan Z.H., Liu X., Song Z., Qiu W. (2020). Efficient Oxidation and Adsorption of As(III) and As(V) in Water Using a Fenton-like Reagent, (Ferrihydrite)-Loaded Biochar. Sci. Total Environ..

[204] Zhu Y., Wei J., Li J. (2023). Biochar-Activated Persulfate Oxidation of Arsenic(III): Nonnegligible Roles of Environmentally Persistent Free Radicals. J. Environ. Chem. Eng..

[205] Yu J., Zhu Z., Zhang H., Chen T., Qiu Y., Xu Z., Yin D. (2019). Efficient Removal of Several Estrogens in Water by Fe-Hydrochar Composite and Related Interactive Effect Mechanism of H_2_O_2_ and Iron with Persistent Free Radicals from Hydrochar of Pinewood. Sci. Total Environ..

[206] Zhu N., Li C., Bu L., Tang C., Wang S., Duan P., Yao L., Tang J., Dionysiou D.D., Wu Y. (2020). Bismuth Impregnated Biochar for Efficient Estrone Degradation: The Synergistic Effect between Biochar and Bi/Bi2O3 for a High Photocatalytic Performance. J. Hazard. Mater..

[207] Chen Y., Duan X., Zhang C., Wang S., Ren N., Ho S.-H. (2020). Graphitic Biochar Catalysts from Anaerobic Digestion Sludge for Nonradical Degradation of Micropollutants and Disinfection. Chem. Eng. J..

[208] Xu H., Han Y., Wang G., Deng P., Feng L. (2022). Walnut Shell Biochar Based Sorptive Remediation of Estrogens Polluted Simulated Wastewater: Characterization, Adsorption Mechanism and Degradation by Persistent Free Radicals. Environ. Technol. Innov..

[209] Wang T., Zheng J., Cai J., Liu Q., Zhang X. (2022). Visible-Light-Driven Photocatalytic Degradation of Dye and Antibiotics by Activated Biochar Composited with K^+^ Doped g-C3N4: Effects, Mechanisms, Actual Wastewater Treatment and Disinfection. Sci. Total Environ..

[210] Shi J., Wang J., Liang L., Xu Z., Chen Y., Chen S., Xu M., Wang X., Wang S. (2021). Carbothermal Synthesis of Biochar-Supported Metallic Silver for Enhanced Photocatalytic Removal of Methylene Blue and Antimicrobial Efficacy. J. Hazard. Mater..

[211] Lian F., Yu W., Zhou Q., Gu S., Wang Z., Xing B. (2020). Size Matters: Nano-Biochar Triggers Decomposition and Transformation Inhibition of Antibiotic Resistance Genes in Aqueous Environments. Environ. Sci. Technol..

[212] Zeng L., Wu M., Wu G.-J. (2019). Electron exchange capacity of rice biochar at different preparation temperatures. China Environ. Sci..

[213] Liu G., Zhang X., Liu H., He Z., Show P.L., Vasseghian Y., Wang C. (2023). Biochar/Layered Double Hydroxides Composites as Catalysts for Treatment of Organic Wastewater by Advanced Oxidation Processes: A Review. Environ. Res..

[214] Xiang L., Liu S., Ye S., Yang H., Song B., Qin F., Shen M., Tan C., Zeng G., Tan X. (2021). Potential Hazards of Biochar: The Negative Environmental Impacts of Biochar Applications. J. Hazard. Mater..

[215] Kharisov B.I., Kharissova O.V. (2019). Carbon Allotropes in the Environment and Their Toxicity. Carbon Allotropes: Metal-Complex Chemistry, Properties and Applications.

[216] El-Naggar A., Lee M.-H., Hur J., Lee Y.H., Igalavithana A.D., Shaheen S.M., Ryu C., Rinklebe J., Tsang D.C.W., Ok Y.S. (2020). Biochar-Induced Metal Immobilization and Soil Biogeochemical Process: An Integrated Mechanistic Approach. Sci. Total Environ..

[217] Cui H., Li D., Liu X., Fan Y., Zhang X., Zhang S., Zhou J., Fang G., Zhou J. (2021). Dry-Wet and Freeze-Thaw Aging Activate Endogenous Copper and Cadmium in Biochar. J. Clean. Prod..

[218] Rombolà A.G., Fabbri D., Baronti S., Vaccari F.P., Genesio L., Miglietta F. (2019). Changes in the Pattern of Polycyclic Aromatic Hydrocarbons in Soil Treated with Biochar from a Multiyear Field Experiment. Chemosphere.

[219] Joseph S., Camps-Arbestain M., Lin Y., Munroe P., Chia C.H., Hook J.M., Zwieten L.V., Kimber S., Cowie A.L., Singh B.P. (2010). An Investigation into the Reactions of Biochar in Soil. Soil Res..

[220] Kim H.-B., Kim S.-H., Jeon E.-K., Kim D.-H., Tsang D.C.W., Alessi D.S., Kwon E.E., Baek K. (2018). Effect of Dissolved Organic Carbon from Sludge, Rice Straw and Spent Coffee Ground Biochar on the Mobility of Arsenic in Soil. Sci. Total Environ..

[221] Jia C., Luo J., Fan J., Clark J.H., Zhang S., Zhu X. (2021). Urgently Reveal Longly Hidden Toxicant in a Familiar Fabrication Process of Biomass-Derived Environment Carbon Material. J. Environ. Sci..

[222] Liu J., Gao N., Wen X., Jia H., Lichtfouse E. (2021). Plant and Algal Toxicity of Persistent Free Radicals and Reactive Oxygen Species Generated by Heating Anthracene-Contaminated Soils from 100 to 600 °C. Environ. Chem. Lett..

[223] Xu Y., Lu X., Su G., Chen X., Meng J., Li Q., Wang C., Shi B. (2023). Scientific and Regulatory Challenges of Environmentally Persistent Free Radicals: From Formation Theory to Risk Prevention Strategies. J. Hazard. Mater..

[224] Jaligama S., Patel V.S., Wang P., Sallam A., Harding J., Kelley M., Mancuso S.R., Dugas T.R., Cormier S.A. (2018). Radical Containing Combustion Derived Particulate Matter Enhance Pulmonary Th17 Inflammation via the Aryl Hydrocarbon Receptor. Part. Fibre Toxicol..

[225] Harmon A.C., Hebert V.Y., Cormier S.A., Subramanian B., Reed J.R., Backes W.L., Dugas T.R. (2018). Particulate Matter Containing Environmentally Persistent Free Radicals Induces AhR-Dependent Cytokine and Reactive Oxygen Species Production in Human Bronchial Epithelial Cells. PLoS ONE.

[226] Reed J.R., dela Cruz A.L.N., Lomnicki S.M., Backes W.L. (2015). Environmentally Persistent Free Radical-Containing Particulate Matter Competitively Inhibits Metabolism by Cytochrome P450 1A2. Toxicol. Appl. Pharmacol..

[227] Chuang G.C., Xia H., Mahne S.E., Varner K.J. (2017). Environmentally Persistent Free Radicals Cause Apoptosis in HL-1 Cardiomyocytes. Cardiovasc. Toxicol..

[228] Zhao S., Miao D., Zhu K., Kelin T., Wang C., Sharma V., Jia H. (2019). Interaction of Benzo[a]Pyrene with Cu(II)-Montmorillonite: Generation and Toxicity of Environmentally Persistent Free Radicals and Reactive Oxygen Species. Environ. Int..

[229] Thevenot P., Saravia J., Jin N., Giaimo J., Chustz R., Mahne S., Kelley M., Hebert V., Dellinger B., Dugas T. (2012). Radical-Containing PM0.2 Initiates Epithelial-to-Mesenchymal Transitions in Airway Epithelial Cells. Am. J. Respir. Cell Mol. Biol..

[230] Zhang X., Gu W., Ma Z., Liu Y., Ru H., Zhou J., Zang Y., Xu Z., Qian G. (2020). Short-Term Exposure to ZnO/MCB Persistent Free Radical Particles Causes Mouse Lung Lesions via Inflammatory Reactions and Apoptosis Pathways. Environ. Pollut..

[231] Balakrishna S., Saravia J., Thevenot P., Ahlert T., Lominiki S., Dellinger B., Cormier S.A. (2011). Environmentally Persistent Free Radicals Induce Airway Hyperresponsiveness in Neonatal Rat Lungs. Part. Fibre Toxicol..

[232] Huang H.-S., Ma M.-C., Chen J., Chen C.-F. (2003). Changes in Renal Hemodynamics and Urodynamics in Rats with Chronic Hyperoxaluria and after Acute Oxalate Infusion: Role of Free Radicals. Neurourol. Urodyn..

[233] Reinke L.A., Moore D.R., Nanji A.A. (2000). Pronounced Hepatic Free Radical Formation Precedes Pathological Liver Injury in Ethanol-Fed Rats. Alcohol. Clin. Exp. Res..

[234] Burn B.R., Varner K.J. (2015). Environmentally Persistent Free Radicals Compromise Left Ventricular Function during Ischemia/Reperfusion Injury. Am. J. Physiol.-Heart Circ. Physiol..

[235] Wang P., You D., Saravia J., Shen H., Cormier S.A. (2013). Maternal Exposure to Combustion Generated PM Inhibits Pulmonary Th1 Maturation and Concomitantly Enhances Postnatal Asthma Development in Offspring. Part. Fibre Toxicol..

[236] Guan X., Truong L., Lomnicki S., Tanguay R., Cormier S. (2021). Developmental Hazard of Environmentally Persistent Free Radicals and Protective Effect of TEMPOL in Zebrafish Model. Toxics.

[237] Zhang Y., Guo X., Si X., Yang R., Zhou J., Quan X. (2019). Environmentally Persistent Free Radical Generation on Contaminated Soil and Their Potential Biotoxicity to Luminous Bacteria. Sci. Total Environ..

[238] Zhang X., Chen Y., Hu D., Zhao L., Wang L., Wu M. (2019). Neurotoxic effect of environmental persistent free radicals in rice biochar to Caenorhabditis elegans. China Environ. Sci..

[239] Stephenson E.J., Ragauskas A., Jaligama S., Redd J.R., Parvathareddy J., Peloquin M.J., Saravia J., Han J.C., Cormier S.A., Bridges D. (2016). Exposure to Environmentally Persistent Free Radicals during Gestation Lowers Energy Expenditure and Impairs Skeletal Muscle Mitochondrial Function in Adult Mice. Am. J. Physiol. -Endocrinol. Metab..

[240] Lee G.I., Saravia J., You D., Shrestha B., Jaligama S., Hebert V.Y., Dugas T.R., Cormier S.A. (2014). Exposure to Combustion Generated Environmentally Persistent Free Radicals Enhances Severity of Influenza Virus Infection. Part Fibre Toxicol.

[241] Briedé J.J., Godschalk R.W.L., Emans M.T.G., de Kok T.M.C.M., van Agen E., van Maanen J.M.S., van Schooten F.-J., Kleinjans J.C.S. (2004). In Vitro and In Vivo Studies on Oxygen Free Radical and DNA Adduct Formation in Rat Lung and Liver during Benzo[a]Pyrene Metabolism. Free Radic. Res..

[242] Balakrishna S., Lomnicki S., McAvey K.M., Cole R.B., Dellinger B., Cormier S.A. (2009). Environmentally Persistent Free Radicals Amplify Ultrafine Particle Mediated Cellular Oxidative Stress and Cytotoxicity. Part. Fibre Toxicol..

