# Supplementary material for: Biochar-Derived Persistent Free Radicals: A Plethora of Environmental Applications in a Light and Shadows Scenario
Source: Toxics. 2024 Mar 27;12(4):245. doi: 10.3390/toxics12040245 (PMC11054495; doi:10.3390/toxics12040245)
Supplement: Supplementary file 1 [file toxics-12-00245-s001.zip › toxics-2907143-supplementary.pdf]

toxics

Supplementary Materials

Biochar-Derived Persistent Free Radicals: A Plethora of Environmental Applications in a Lights and Shadows Scenario

Silvana Alfei[1,*] and Omar Ginoble Pandoli[1,2]

[1] Department of Pharmacy (DIFAR), University of Genoa, Viale Cembrano, 4, 16148 Genoa, Italy, omar.ginoble-pandoli@unige.it or omarpandoli@puc-rio.br (O.G.P.) (ORCID ID: 0000-0002-2220-7817)
[2] Department of Chemistry, Pontifical Catholic University, Rua Marquês de São Vincente, 225, Rio de Janeiro 22451-900, Brazil
*Correspondence: alfei@difar.unige.it (S.A.) (ORCID ID: 0000-0002-4630-4371)

Table S1. Main sources and general application of BCs.

Source biomass	Ref. *	Applications	Refs.
Crop residue	[53]	Carbon sequestration	[67]
Kitchen waste	[54]		
Forestry	[55]	Soil amendment	[68]
Agricultural waste	[56]		
Sugar beet tailings	[57]	Composting	[69]
Forest residues	[58]		
Waste wood	[59]	Wastewater treatment	[70]
Bioenergy crops	[60]		
Municipal solid waste	[61]	Energy production/storage	[71]
Wheat straw	[62]	Adsorbing xenobiotics	[72]
Rice straw	[63]	Reducing greenhouse emission gas	[73]
Food manure	[64]	Xenobiotics degradation	[74]
Animal manure	[65]	Catalysis	[75]
Corn cob	[66]		

* The references in the Table correspond to those reported in the main text.

Table S2. Fast and slow pyrolysis details.

	Fast Pyrolysis	Slow Pyrolysis	Ref.
**Target Product**	Bio-oils	Biochar	
**Reactors**	Bubbling fluidized bed<br>Ablative reactor<br>Rotary cone	Fixed bed pyrolysis reactor<br>Auger pyrolysis reactor	
**Warming rate**	10-10000°C/min	0.1-10°C/min	
**RT at ⇧ temperature**	0.5-2 seconds	> 1 hour	
**Aeration**	Oxygen-free	Oxygen-free or limited	
**Advantages**	⇧ Yield of bio-oil	⇧ Yield of BC<br>Accepted a wide range of particle size	[97]
**Disadvantages**	⇩ BC yield<br>Required fine particles of biomass feed (1-2 mm)<br>Prefer biomass with low moisture content (<10%)	Further treatment of gases is needed due to high CO concentrations	
**Applications**	⇧ Potential for energy applications	Improvement of soil quality	

* The references in the Table correspond to those reported in the main text. RT = Residence time; ⇧ = high, higher; ⇩ = low, lower.

Table S3. Techniques typically used to characterize BCs in terms of their physicochemical, surface, and structural characterization.

Biochar characterization techniques					
Physicochemical Characterization		Surface Characterization		Structural/Molecular Characterization	
**Chemical**	**Physical**	**Investigation**	**Technique**	**Investigation**	**Technique**
pH	Surface area (BET)	Morphology	SEM	Thermal behavior	TGA
Cation exchange capacity (CEC)	Size (nm) (TEM)	Functional groups	FTIR/Raman	Structural arrangements	XRD
Electrical conductivity	Bulk density	Surface elements	SEM, EDXS, XFR	Aromaticity	NMR/Raman
	Pore size (BET)	Surface oxygen	Boehm titration	Free radicals	EPR/ESR

SEM = Scanning Electron Microscopy; TEM = transmission electron microscopy analysis; EDXS = Energy-Dispersive X-ray Spectroscopy (EDXS); XFR = X-ray Fluorescence spectroscopy; FTIR = Fourier Transform Infrared Spectrometer; TGA = thermos-gravimetric analysis; XRD = X-Ray Diffraction; BET = Brunauer-Emmett-Teller (BET); NMR = Nuclear Magnetic Resonance (NMR); Raman = Raman Spectroscopy; Cation Exchange Capacity (CEC); EPR = Electron Paramagnetic Resonance; ESR = Electron Spin Resonance (ESR) spectroscopy.

Table S4. Properties of BCs produced from various feedstocks at various production temperatures. The table has been constructed on a literature example [99].

Feedstock	Pyrolysis temp.	Yield	Ash	pH	C	H	O	N	Surface area
	(°C)	(%)	(%)		(%)	(%)	(%)	(%)	(m^2 g^{-1})
Canola straw	400	27.4	–	–	45.7	–	–	0.19	–
Corn cobs	500	18.9	13.3	7.8	77.6	3.05	5.11	0.85	0.0
Corn stover	450	15.0	58.0	–	33.2	1.40	8.60	0.81	12.0
Corn stover	500	17.0	32.8	7.2	57.3	2.86	5.45	1.47	3.1
Cottonseed hull	200	83.4	3.1	–	51.9	6.00	40.5	0.60	–
Cottonseed hull	800	24.2	9.2	–	90.0	0.60	7.00	1.90	322.0
Fescue straw	100	99.9	6.9	–	48.6	7.25	44.1	0.64	1.8
Fescue straw	700	28.8	19.3	–	94.2	1.53	3.60	0.70	139.0
Oak bark	450	–	11.1	–	71.2	2.63	12.9	0.46	1.9
Oakwood	400–450	–	2.9	–	82.8	2.70	8.05	0.31	2.7
Orange peel	150	82.4	0.5	–	50.6	6.20	41.0	1.75	22.8
Orange peel	700	22.2	2.8	–	71.6	1.76	22.2	1.72	201.0
Peanut shell	300	36.9	1.2	7.8	68.27	3.85	25.89	1.91	3.1
Peanut shell	700	21.9	8.9	10.6	83.76	1.75	13.34	1.14	448.2
Peanut straw	400	28.2	–	–	42.90	–	–	1.50	–
Pine needles	100	91.2	1.1	–	50.87	6.15	42.27	0.71	0.7
Pine needles	700	14.0	2.2	–	86.51	1.28	11.08	1.13	490.8
Pine shaving	100	99.8	1.2	–	50.60	6.68	42.70	0.05	1.6
Pine shaving	700	22.0	1.7	–	92.30	1.62	6.00	0.08	347.0
Pinewood	700	–	38.8	6.6	95.30	0.82	3.76	0.12	29.0
Poplar wood	400	32.0	3.5	9.0	67.30	4.42	–	0.78	3.0
Rice husk	500	–	42.2	–	42.10	2.20	12.10	0.50	34.4
Saw dust	450	–	1.1	5.9	72.00	3.50	24.41	0.08	–
Saw dust	550	–	2.8	12.1	85.00	1.00	13.68	0.30	–

Soybean stover	300	37.0	10.4	7.3	68.81	4.29	24.99	1.88	5.6
Soybean stover	700	21.6	17.2	11.3	81.98	1.27	15.45	1.30	420.3
Soybean straw	400	24.7	–	–	44.10	–	–	2.38	–
Spruce wood	400	36.0	1.9	6.9	63.50	5.48	–	1.02	1.8
Spruce wood	525	–	4.7	8.6	78.30	3.04	–	1.17	40.4
Wheat straw	400	34.0	9.7	9.1	65.70	4.05	–	1.05	4.8
Wheat straw	525	–	12.7	9.2	74.40	2.83	–	1.04	14.2
Chicken litter	620	43-49	53.2	-	41.50	1.20	0.70	2.77	-
Poultry litter	350	54.3	30.7	8.7	51.07	3.79	15.63	4.45	3.9
Poultry litter	700	36.7	46.2	10.3	45.91	1.98	10.53	2.07	50.9
Tire rubber	200	93.5	15.0	-	74.70	6.38	3.92	-	-
Tire rubber	800	43.0	10.5	-	86.0	0.87	2.16	0.47	50.0

Disclaimer/Publisher's Note: The statements, opinions and data contained in all publications are solely those of the individual author(s) and contributor(s) and not of MDPI and/or the editor(s). MDPI and/or the editor(s) disclaim responsibility for any injury to people or property resulting from any ideas, methods, instructions or products referred to in the content.
